# Food insecurity, type 2 diabetes, and hyperglycaemia: A systematic review and meta‐analysis

**DOI:** 10.1002/edm2.315

**Published:** 2021-11-02

**Authors:** Sourik Beltrán, Daniel J. Arenas, Marissa Pharel, Canada Montgomery, Itzel Lopez‐Hinojosa, Horace M. DeLisser

**Affiliations:** ^1^ Department of Medicine Massachusetts General Hospital Boston Massachusetts USA; ^2^ Perelman School of Medicine University of Pennsylvania Philadelphia Pennsylvania USA; ^3^ Rush Medical College Rush University Chicago Illinois USA; ^4^ Pritzker School of Medicine University of Chicago Chicago Illinois USA

**Keywords:** food insecurity, social determinants of health, type 2 diabetes

## Abstract

**Aims:**

Food insecurity (FIS) is a major public health issue with possible implications for type 2 diabetes mellitus (T2DM) risk. We conducted a systematic review and meta‐analysis to explore the association between FIS and T2DM.

**Methods:**

We performed a systematic search in PubMed, Embase, Scopus, and Web of Science. All cross‐sectional, peer‐reviewed studies investigating the link between FIS and T2DM were included. Population characteristics, study sizes, covariates, T2DM diagnoses, and diabetes‐related clinical measures such as fasting blood glucose (FBG) and HbA1c were extracted from each study. Outcomes were compared between food insecure and food secure individuals. Effect sizes were combined across studies using the random effect model.

**Results:**

Forty‐nine peer‐reviewed studies investigating the link between FIS and T2DM were identified (*n* = 258,250). Results of meta‐analyses showed no association between FIS and clinically determined T2DM either through FBG or HbA1c: OR = 1.22 [95%CI: 0.96, 1.55], Q(df = 5) = 12.5, *I* ^2^ = 60% and OR = 1.21 [95%CI: 0.95, 1.54], Q(df = 5) = 14; *I* ^2^ = 71% respectively. Standardized mean difference (SMD) meta‐analyses yielded no association between FIS and FBG or HbA1c: *g* = 0.06 [95%CI: −0.06, 0.17], Q(df = 5) = 15.8, *I* ^2^ = 68%; *g* = 0.11 [95% CI: −0.02, 0.25], Q(df = 7) = 26.8, *I* ^2^ = 74% respectively. For children, no association was found between FIS and HbA1c: *g* = 0.06 [95%CI: 0.00, 0.17], Q(df = 2) = 5.7, *I* ^2^ = 65%.

**Conclusions:**

Despite multiple proposed mechanisms linking FIS to T2DM, integration of the available literature suggests FIS is not associated with clinically determined T2DM or increases in FBG or HbA1c among adult patients.

## Graphical abstract

Despite potential mechanisms linking food insecurity (FIS) to type 2 diabetes mellitus (T2DM), integration of the available literature suggests FIS is not associated with clinically determined T2DM or increases in fasting blood sugar or HbA1c among adult patients.

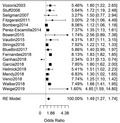

## 1 INTRODUCTION

Dietary recommendations play an essential role in the prevention and management of type 2 diabetes mellitus (T2DM).^1^ Diets high in fresh vegetables and fruits as well as those containing low‐glycaemic carbohydrates (eg the ‘Mediterranean diet’) have been shown across many studies to result in significant improvements in glucose tolerance.^2^, ^3^ Nevertheless, fostering consistent adherence to diet modifications in T2DM care remains a major challenge for both patients and clinicians.^4^, ^5^

Food insecurity (FIS), defined as improper or inconsistent access to high‐quality or nutritious foods, is a major public health problem affecting upwards of 2 billion people worldwide as of 2020.^6^, ^7^ It has been increasingly recognized as an structural barrier that can significantly affect patients’ ability to adhere to clinical dietary recommendations.^7^ Given the relevance of diet across a wide range of health domains, studies have demonstrated that FIS is associated with various health issues including obesity, depression, anxiety, and cognitive impairment.^8^, ^9^, ^10^ Furthermore, FIS has been associated with significantly higher incidence of self‐reported stress, worse self‐perceived health, and poorer quality of life.^11^, ^12^, ^13^, ^14^

There are at least two mechanisms that could provide a link between FIS and T2DM. The first is that inadequate access to healthy and fresh foods, particularly in areas known as food deserts, may lead individuals with FIS to rely on cheaper food products that contain higher proportions of salt, unhealthy fats, and processed carbohydrates as well as lower quantities of dietary fibre.^15^, ^16^, ^17^, ^18^ Over time, this continued exposure to processed foods may lead to the development of T2DM. A second mechanism relates to chronic stress. As FIS is strongly associated with worse self‐reported quality of life and higher levels of stress and anxiety, it is also possible that the generalized toxic stress associated with FIS may itself lead to increased activation of cortisol release pathways which could in turn worsen derangements in glucose tolerance and insulin sensitivity.^19^, ^20^

Numerous studies have attempted to explore the relationship between FIS and T2DM. In an initial systematic review and meta‐analysis in 2019, Abdurahman et al. sought to integrate the existing data, reporting an overall positive association between FIS and T2DM across 17 cross‐sectional adult studies.^21^ Their results, however, may not have fully captured the relationship between FIS and T2DM. Fifteen of the 17 included studies involved self‐reported diagnosis (rather than a clinically determined one), a confounding factor identified in other meta‐analyses related to FIS,^22^ and relationships between FIS and objective measures of glucose intolerance (eg blood glucose, HbA1c) were not explored. Therefore, we conducted a further, more extensive, systematic review and meta‐analysis to characterize more thoroughly the possible relationship between FIS and T2DM as well as investigate the effect of FIS on objective diabetic markers.

## 2 METHODS

### 2.1 Data sources

This systematic review and meta‐analysis is the result of a broad, systematic search of the literature to investigate the link between FIS and cardiometabolic disease. A broad search of multiple cardiovascular risk factors was pursued with the goal of obtaining data relevant to the association between FIS and T2DM from studies whose primary aim may have been a separate measure of cardiovascular risk. The effectiveness of this approach has been demonstrated in prior reviews.^8^, ^22^ The original search took place on September 9, 2019, and involved four major databases: PubMed, Scopus, Embase, and Web of Science. The search was registered in Prospero on January 28, 2020 (registration no. CRD42020149560). Exact search terms included in the initial literature search are presented in the Supplementary Information. All peer‐reviewed human studies of any population, methodology, or publication year were included in the search using the Medical Subject Headings pertaining to FIS as well as those of hypertension, metabolic syndrome, dyslipidaemia, and T2DM.

### 2.2 Study selection

Each abstract identified in the initial search was randomized and individually screened through Abstrackr.^23^ Four authors inspected each abstract to determine relevance as defined by the following criteria: (a) the study involved FIS or a comparable concept; (b) the study involved hypertension, metabolic syndrome, dyslipidaemia, or T2DM; (c) the study presented primary, quantitative data; and (d) the study was peer‐reviewed and published in or translated to English. Only studies which met all four criteria were included. Inter‐rater reliability was assessed using the Fleiss’ kappa tool for measuring group inter‐rater agreement as well as agreement between rater‐pairs.^22^, ^24^ Articles that were excluded by all four authors were automatically discarded; those with approval from at least one author were further discussed and reviewed by an additional author. These studies underwent full text evaluation by at least two authors for the presence of primary data directly investigating an association between FIS and T2DM. Disagreements between reviewing authors were addressed and resolved by an additional author. Finally, studies that were approved for inclusion based on full text evaluation were then assessed by three authors for individual study bias using the AXIS tool for quality assessment of cross‐sectional studies.^25^

### 2.3 Data extraction

Each study that was identified through the above process underwent extraction of the following information: population characteristics, sample size, design, measures, and all available outcomes related to FIS and T2DM. The data extracted at this stage were grouped by specific outcome and yielded four major areas of inquiry: (a) the association between FIS and self‐reported T2DM, (b) the association between FIS and clinically diagnosed T2DM, (c) the association between FIS and HbA1c, and (d) the association between FIS and fasting blood glucose (FBG). Based on these available data, meta‐analyses were deemed possible for odds ratio (OR) and standardized mean differences (SMD, eg Hedges' *g*). Effect sizes were extracted by one author directly or manually calculated using the study's primary data. For studies that reported data using multiple categories of FIS (eg mild or moderate), categories were merged either by pooling primary data or by combining multiple effect sizes through the random effects (RE) model.^26^ Cutoffs used for FBG and HbA1c definitions of T2DM were those provided by individual studies. The specific cutoffs and definitions for each study are shown in the Tables S1‐S3. FBG cutoffs for T2DM were consistently 126 mg/dl while HbA1c cutoffs ranged from 6.5% to 9%.

### 2.4 Data synthesis and meta‐analysis

For the primary areas of inquiry described above, meta‐analyses were only conducted if three or more studies were available which reported a particular outcome measurement. Analysis was conducted using the metafor package in R based on the RE size model (this does not assume populations from which samples were derived have identical probability distributions).^27^ Meta‐analyses were calculated using the DerSimonian‐Laird estimator.^28^ For meta‐analyses of OR data, the logarithm was used as the effect size. Studies used in each meta‐analysis were assessed for heterogeneity by calculating total variance (Q), degrees of freedom (df), and the *I* ^2^ statistic.^29^ Studies were assessed for publication bias by testing for significant funnel‐plot‐asymmetry using the Begg‐Mazumdar rank correlation test and the Egger's regression test.^30^, ^31^

## 3 RESULTS

### 3.1 Study characteristics

Following the initial literature search, a total 787 abstracts were identified which were individually reviewed by four authors using the relevance criteria. Assessment of inter‐rater reliability yielded significant agreement between the four authors: Fleiss' kappa = 0.68 [95%CI: 0.66, 0.71, *N* = 4], a score generally regarded as indicative of ‘good’ or ‘substantial’ agreement based on available guidelines.^32^ Additionally, every permutation of author pairs showed inter‐rater agreements above 0.6. At this stage, 196 studies remained, each of which underwent full text evaluation by four authors using the above inclusion criteria. Text evaluation resulted in a total of 82 studies containing primary data investigating the association between FIS and T2DM. Among the 82 studies, 33 were excluded for containing either unusable or incomplete primary data (*N* = 30) or for reporting data that overlapped with other studies (*N* = 3). The final cohort consisted of a total of 49 cross‐sectional studies containing primary data on the association of FIS with T2DM for adults and children across eight countries (USA, Canada, Mexico, Portugal, Malaysia, Iran, Ecuador, and Australia). The study selection process is summarized in Figure S1.

Of the final cohort of 49 studies, 46 presented data on adults (combined *n* = 258,250)^13^, ^33^, ^34^, ^35^, ^36^, ^37^, ^38^, ^39^, ^40^, ^41^, ^42^, ^43^, ^44^, ^45^, ^46^, ^47^, ^48^, ^49^, ^50^, ^51^, ^52^, ^53^, ^54^, ^55^, ^56^, ^57^, ^58^, ^59^, ^60^, ^61^, ^62^, ^63^, ^64^, ^65^, ^66^, ^67^, ^68^, ^69^, ^70^, ^71^, ^72^, ^73^, ^74^, ^75^, ^76^, ^77^ and three presented data on children (combined *n* = 10,280).^78^, ^79^, ^80^ For adults, it was deemed that five meta‐analyses were possible: (a) ORs of FIS and self‐reported T2DM, (b) ORs of FIS and T2DM determined by FBG, (c) ORs of FIS and T2DM determined by HbA1c, (d) SMD of FBG, and (e) SMD of HbA1c. For children, only one meta‐analysis was possible: SMD of HbA1c. For both children and adults, insufficient subgroup data was presented which could allow for analysis by sample demographics. Precise grouping and individual study characteristics for each meta‐analysis can be found in Tables S1–S11.

### 3.2 Adult studies

**FIGURE 1:**
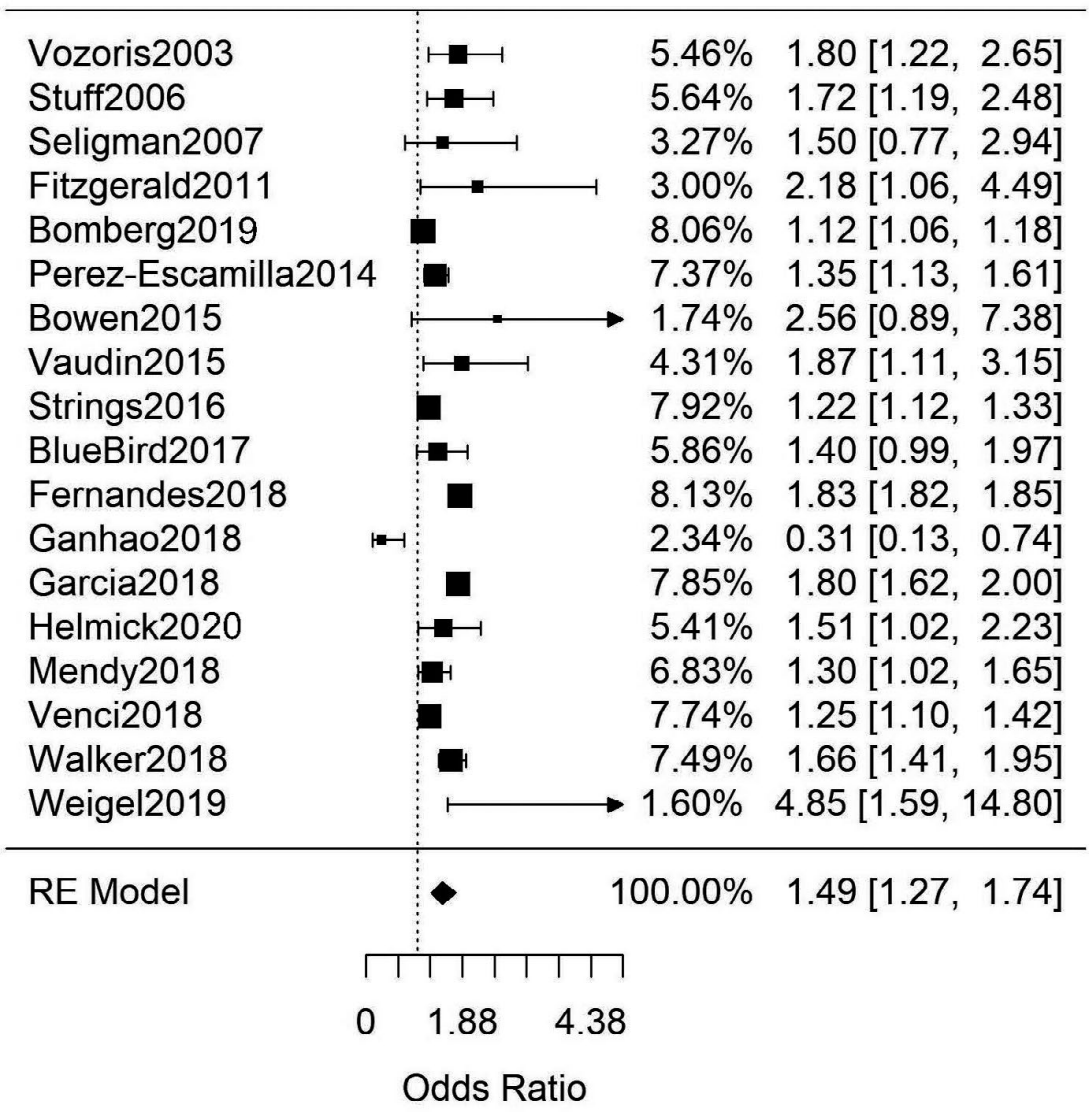
Meta‐analysis of adult studies investigating the association between FIS and self‐reported T2DM

Among the 46 adult studies included in this systematic review, 18 studies (Table S1) contained primary data on the association between FIS and self‐reported T2DM (combined *n* = 182,542).^33^, ^34^, ^35^, ^36^, ^37^, ^38^, ^39^, ^40^, ^41^, ^42^, ^43^, ^44^, ^45^, ^46^, ^47^, ^48^, ^49^, ^50^ Given previously noted differences in results for studies measuring self‐reported versus clinically determined chronic disease among food insecure patients,^22^ these 18 studies were analysed separately from studies which involved clinical diagnoses of T2DM. Meta‐analysis of this group yielded a significant, combined OR of 1.49 [95%CI: 1.27, 1.74; *n* = 182,542; Q(df = 17) = 462.6; *I* ^2^ = 96%] (Figure 1). Notably, there was evidence of funnel‐plot‐asymmetry: a potential publication bias in favour of smaller‐sample studies with positive effect sizes (Figure S2).

**FIGURE 2:**
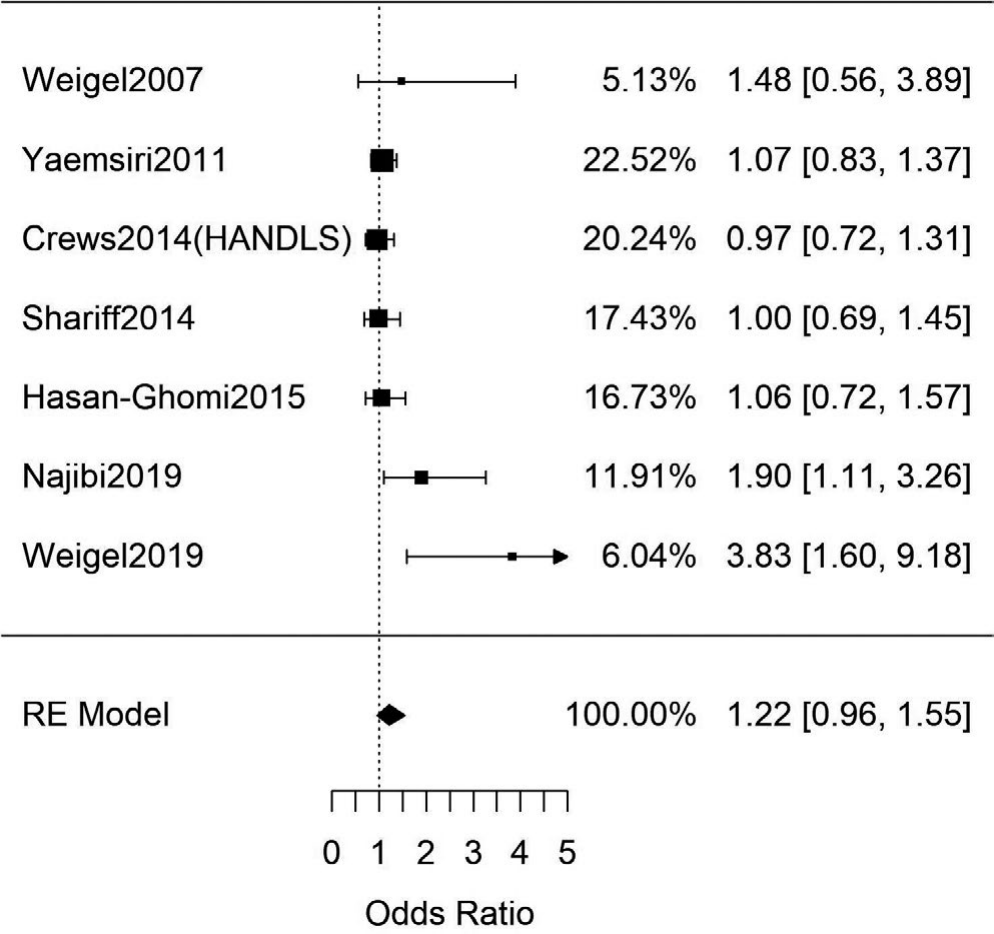
Meta‐analysis of adult studies investigating FIS and T2DM as determined by FBG measurements

**FIGURE 3:**
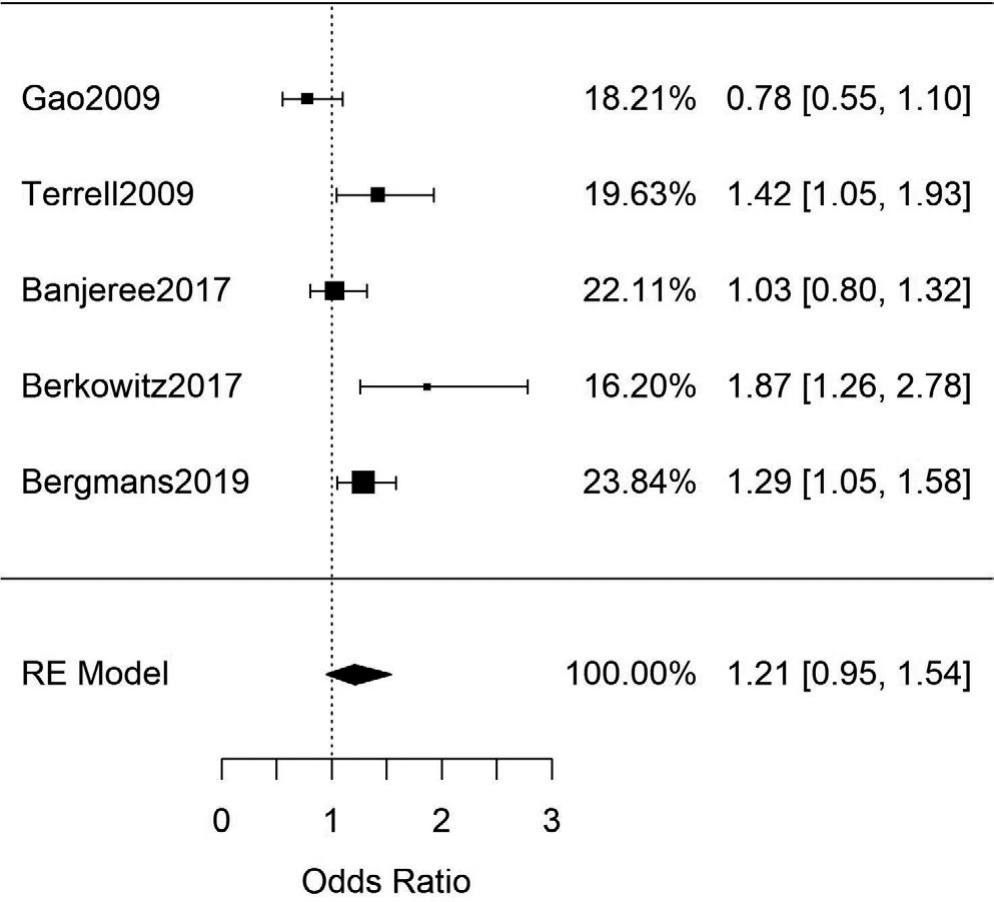
Meta‐analysis of adult studies investigating FIS and T2DM as determined by HbA1c measurements

Next, studies that investigated the association between FIS and clinically diagnosed T2DM were analysed. Seven studies (Table S2) reported data in which T2DM diagnoses were established through FBG measurements (combined *n* = 8390)^50^, ^51^, ^52^, ^53^, ^54^, ^55^, ^56^ while five studies (Table S3) reported T2DM diagnoses established through HbA1c measurements (combined *n* = 25,368).^57^, ^58^, ^59^, ^60^, ^61^ Meta‐analyses of these two groups both showed no significant association between FIS and clinically diagnosed T2DM through either FBG or HbA1c measurements (OR = 1.22 [95%CI: 0.96, 1.55; *n* = 8390; Q(df = 5) = 12.5; *I* ^2^ = 60%], Figure 2; and OR = 1.21 [95%CI: 0.95, 1.54; *n* = 8390; Q(df = 5) = 14; *I* ^2^ = 71%], Figure 3; respectively). There was evidence of funnel‐plot‐asymmetry and potential publication bias for the meta‐analysis for FIS and FBG (Figure S3), but none for the meta‐analysis involving FIS and HbA1c measurements (Figure S4).

**FIGURE 4:**
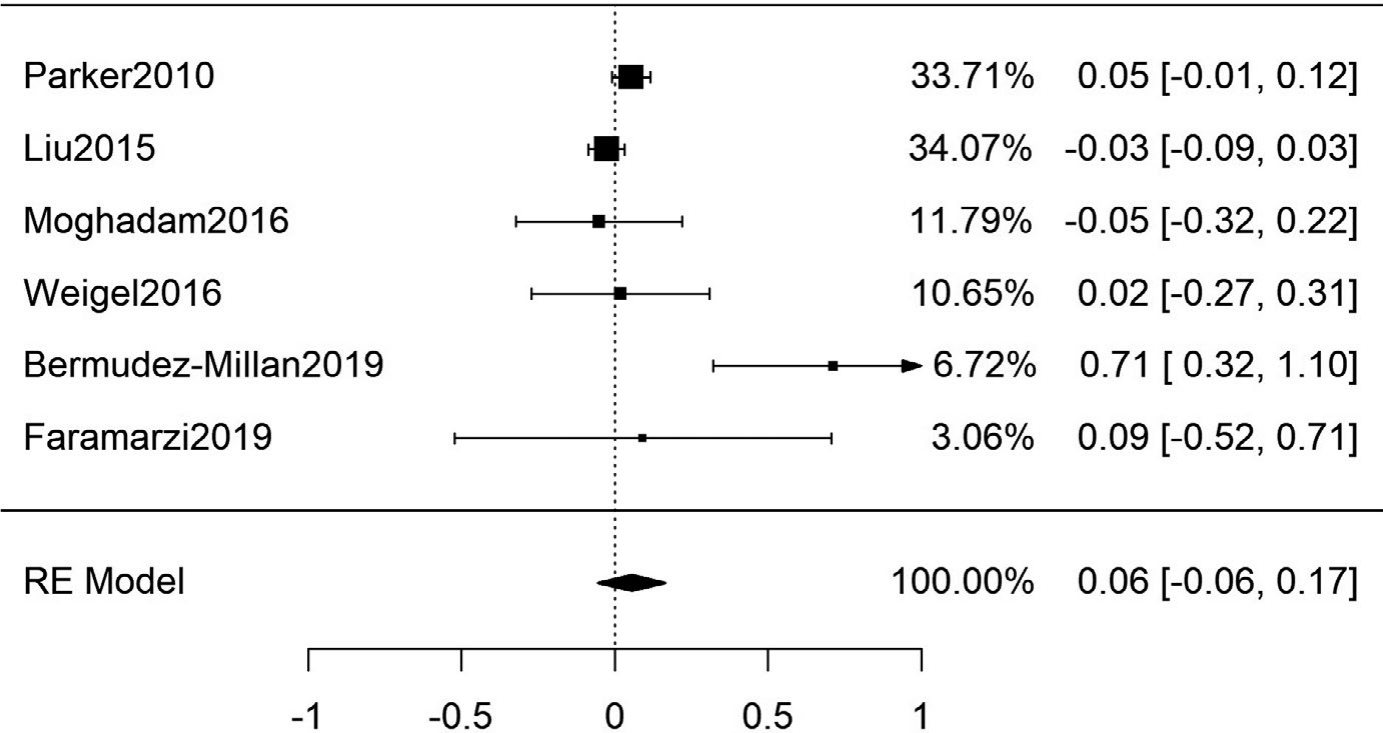
Meta‐analysis of SMD for FIS and FBG among adult patients

**FIGURE 5:**
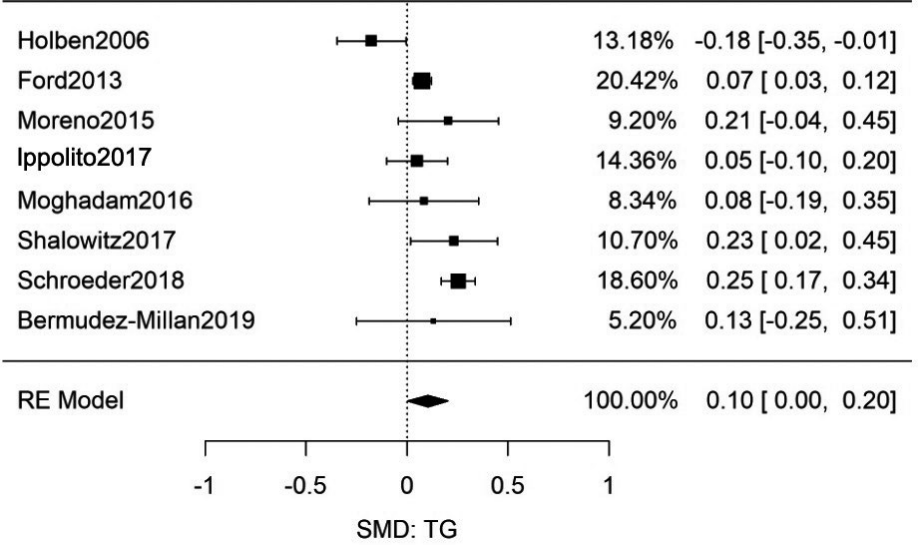
Meta‐analysis of SMD for FIS and HbA1c among adult patients

Six studies (Table S4) were found to contain primary data sufficient to calculate the SMD of FBG between patients with and without FIS (combined *n* = 12,455).^13^, ^62^, ^63^, ^64^, ^65^, ^66^ Results of this meta‐analysis yielded no significant difference in FBG between patients with or without FIS: (*g* = 0.06 [95%CI: −0.06, 0.17; *n* = 12,455; Q(df = 5) = 15.8; *I* ^2^ = 68%], Figure 4); there was no significant funnel‐plot‐asymmetry (Figure S5). An additional eight studies (Table S5) were found to contain data that allowed the evaluation of the SMD for HbA1c for patients with and without FIS (combined *n* = 16,348).^13^, ^64^, ^67^, ^68^, ^69^, ^70^, ^71^, ^72^ This meta‐analysis also showed no significant difference in HbA1c between FIS and food secure individuals (*g* = 0.11 [95% CI: −0.02, 0.25; *n* = 16,348; Q(df = 7) = 26.8; *I* ^2^ = 74%], Figure 5) and no significant funnel‐plot‐asymmetry (Figure S6).

Six adult studies reported data that was insufficient or inadequate for meta‐analysis (Tables S6 and S7). Three of these studies reported beta coefficient for HbA1c between patients with and without FIS (combined *n* = 1303).^73^, ^74^, ^75^ Due to differences in the presented data, beta coefficient meta‐analysis was not possible. Results of two of these studies found significant associations between food insecurity and HbA1c: *β* = 0.51 and *β* = 0.12 respectively.^73^, ^74^ The third study found no association: *β* = 0.092.^75^ One US and one Canadian study reported ORs for T2DM between patients with and without FIS using health record‐documented T2DM.^76^, ^77^ Results of each showed no association between FIS and documented T2DM: OR = 0.89 [95%CI: 0.79, 1.02] for Wang et al.^76^ and AOR = 2.04 [95%CI: 0.99, 4.26] for Tait et al.^77^ Lastly, Crews et al. (represented in an above meta‐analysis) reported separate data on the relationship between FIS and T2DM determined by non‐fasting blood glucose and found no significant association.^53^

### 3.3 Paediatric studies

Only three paediatric studies were included in the final cohort of this systematic review (Table S8).^78^, ^79^, ^80^ For these three studies, investigation of the SMD for HbA1c demonstrated no significant difference in HbA1c measurements between paediatric patients with or without FIS (*g* = 0.06 [95%CI: 0.00, 0.17; *n* = 16,348; Q(df = 2) = 5.7; *I* ^2^ = 65%, Figure S7]). Assessment for publication bias in this group was negative (Figure S8).

### 3.4 Subgroup analyses

An insufficient number of studies reported subgroup‐level data that would allow for meta‐analysis based on patient populations or other demographic factors. However, subgroup meta‐analyses of (a) only adjusted odds ratios for FIS and T2DM determined by FBS (Table S9; Figures S9 and S10); (b) only unadjusted odds ratios for FIS and T2DM determined by HbA1c (Table S10; Figures S11 and S12); and (c) odds ratios for FIS and T2DM using HbA1c cut off 7% (Table S11; Figures S13 and S14) all demonstrated non‐significant findings.

### 3.5 Risk of bias within individual studies

Evaluation of the individual included studies through the AXIS tool did not reveal concerning results that would warrant study exclusion. An in‐depth discussion of the findings of individual study assessment by the AXIS tool can be found in the Supporting Information.

## 4 DISCUSSION

FIS is a major public health issue with numerous documented health effects.^6^, ^7^, ^8^, ^9^, ^10^, ^11^, ^12^, ^13^, ^14^ Given the importance of dietary habits and chronic stress in the development of metabolic disease, it is reasonable to infer that FIS could lead to elevations in glycaemic markers and increased risk for T2DM.^15^, ^16^, ^17^, ^18^, ^19^, ^20^ Such associations would have clinical relevance as providers could consider more widespread use of FIS screening tools to better evaluate patients' individual risk for developing T2DM. From a public health perspective, such findings could help to justify FIS‐based interventions with the added goal of reducing the population‐level burden of T2DM. Nevertheless, the results of this review suggest that FIS is not associated with either clinically diagnosed T2DM or significant differences in FBG or HbA1c. The latter findings are particularly meaningful as a proposed link between FIS‐driven poor (ie high‐glycaemic) dietary habits and T2DM would be expected to be demonstrated by differences in FBG or HbA1c measurements between patients with and without FIS.

This systematic review and meta‐analysis identified 49 cross‐sectional studies (combined *n* = 258,250) containing primary data on the association between FIS and T2DM. Our results showed that FIS is not associated with clinically diagnosed T2DM for adults when FBG or HbA1c measurements are used to establish a diagnosis. SMD meta‐analyses found no significant differences in FBG or HbA1c levels between food insecure and food secure adults. Instead, similar to a prior review by the authors,^22^ this study demonstrated that FIS is specifically associated with increased self‐reported T2DM among adults. For paediatric patients, SMD meta‐analysis found no significant difference in HbA1c levels between paediatric patients with and without FIS.

There are several explanations that may account for the results of this review. First, it is important to recognize that FIS is a complex issue which may show significant heterogeneity depending on differences in contributing factors such as social, economic, or geographic considerations.^81^, ^82^, ^83^ For example, individuals in one setting may experience FIS as a lack of available fresh and healthy foods. This could lead to increased dietary intake of lower‐quality, high‐calorie foods which could increase a person's risk for T2DM. In contrast, individuals in another context could experience FIS as hunger or chronic starvation where quantity, rather than quality, is the primary driver. As a separate mechanism, FIS experienced as starvation could suggest an overall hypocaloric diet which itself has been shown to be protective against metabolic disease.^84^, ^85^ Therefore, as the available literature was not found to contain sufficient granularity to assess context‐specific differences in FIS, the overall non‐significant effect sizes observed in this review may be the result of multiple, competing mechanisms whose impacts on patients’ risk for T2DM are obscured in aggregate. This explanation is further corroborated by the moderate to high heterogeneity observed in each meta‐analysis as quantified by the *I* ^2^ statistic.

Next, it is noteworthy that for both children and adults, SMD meta‐analysis of HbA1c yielded similar, near‐significant results with wide CIs and relatively high effect sizes. Although these remain non‐significant findings, the wide CIs observed in each measure suggest that a significant result could be obtained from additional studies contributing to a greater combined sample size. Nevertheless, the other non‐significant findings in this review, in particular the finding of no association between FIS and HbA1c‐determined T2DM, strengthen the view that FIS may not be a substantial contributor to differences in HbA1c or the prevalence T2DM on a population scale.

This review found that FIS was associated with increased self‐reported T2DM, a finding that should be viewed in the context of the publication bias that was identified for this meta‐analysis. This finding is consistent with a prior systematic review and meta‐analysis by the authors which demonstrated parallel findings for hypertension without concerns for publication bias.^22^ One possible explanation for this observation may be related to an overall perception of poorer health among food insecure individuals. Prior studies have shown that individuals with FIS have worse self‐perceived health compared with food secure counterparts.^9^, ^86^ This may be related to associations of FIS with anxiety or depression, increased stress, and overall lower self‐reported quality of life.^8^, ^11^, ^12^, ^13^ It is possible that food insecure patients may be more likely to perceive their health to be poor and therefore may be more likely to report T2DM even in the absence of an established diagnosis. Importantly, this result also highlights the potential unreliability of self‐reported clinical data in population health studies.

It is important to acknowledge that our findings differ from the frequently cited systematic review and meta‐analysis by Abdurahman et al.^21^ that reported a positive association between FIS and T2DM in 2019 (which we learned of prior to initiation of our review). There are several possible reasons for the differences in the two studies. First, our review captured the 17 studies identified by Abdurahman et al. along with an additional 32 cross‐sectional studies, which provided us a larger pool of combined data for our meta‐analyses. Second, the majority of the studies cited involved a self‐reported diagnosis (rather than a clinically determined one), a confounding factor identified in other meta‐analyses related to FIS,^22^ which was again noted in the current study. Finally, Abdurahman et al. did not include assessments for differences in FBG or HbA1c measurements based on food security status, measures which would provide more objective insights into the possible metabolic associations of FIS. We would also note that Abdurahman et al. represented the population size of the study by Pérez‐Escamilla et al.^38^ as its study sample, which led to a combined sample of *n* = 55,353,915 rather than a true combined sample of *n* = 150,935.

The findings of our review also contrast with two additional recent systematic reviews by da Silva Miguel et al.^87^ and Vazquez et al.^88^, both of which were identified during the review process and concluded that FIS is associated with T2DM. However, we note that (a) neither study included meta‐analyses; (b) these reviews did not distinguish between self‐reported versus clinically determined T2DM; and (c) Vazquez et al. relied heavily upon the findings of Abdurahman et al. in making their conclusions, without the addition of other available data. Given the limitations of these three studies, and the findings of this report, we believe caution should be exercised in concluding that FIS is associated with T2DM and suggest that the relationship may be more complex than has been previously described.

This systematic review and meta‐analysis has several strengths. First, as this review involved a systematic search of the literature including multiple, peripherally related cardiometabolic markers (including measures related to hypertension, dyslipidaemia, and metabolic syndrome), we believe we identified significantly more sources of primary data than would have been possible had we begun with a narrower search. This is supported by this review's inclusion of multiple studies whose main hypotheses were not related to the association between FIS and T2DM but were found to contain relevant primary data related to FBG and HbA1c measurements upon full text evaluation. This effect has been shown in previous systematic reviews utilizing a similar methodology.^8^, ^22^, ^89^ Furthermore, although many systematic reviews employ only two reviewers of abstracts and articles, the availability and effort of four reviewers, who as a group had a Fleiss kappa considered to be in substantial agreement,^32^ minimized the probability that an article was erroneously excluded. The rigour of our process was further enhanced by the fact that abstracts with at least one approval from one of the four reviewers underwent further review by the evaluators and an additional author. In addition, as this review included all human studies involving any patient population in any region, we were able to analyse a relatively large number of primary studies involving a diverse sample population. Finally, sufficient studies were included to allow for multiple, distinct meta‐analyses related to FIS and T2DM (clinical disease as well as objective measurements of hyperglycaemia), thus lending increased validity to the overall non‐significant findings of this review.

This review also has important limitations. First, as this systematic review identified only cross‐sectional studies, conclusions cannot be drawn about longitudinal relationships between FIS and T2DM or hyperglycaemia. As dietary habits as well as chronic stress are known to have long‐term effects on the pathophysiology of chronic diseases like T2DM, the paucity of longitudinal data on the association between FIS and T2DM makes it difficult to distinguish if factors like duration of FIS influence individual risk for T2DM. Second, despite the total number of studies included in this review, few studies reported subgroup‐level data on the association between FIS and T2DM. Thus, we were unable to assess the role of FIS on patients' risk of T2DM in subpopulations based on factors like race, ethnicity, or geography. Therefore, in the absence of further subgroup analyses, the heterogeneity of the included studies presents a significant limitation and challenge for the interpretation of the results of this meta‐analysis. As FIS is a complex variable, it is possible that further analysis in other countries, as well as subgroup analysis in specific populations within the US and other better represented countries in the literature, may elucidate significant and more specific associations between food insecurity and diabetes. This review therefore highlights the need for more subpopulation studies, including studies considering covariates such as race, ethnicity and nationality as well as other factors such as depression, anxiety, and the quality of the diet.

Finally, regarding publication bias, it is noteworthy that several funnel‐plot‐asymmetry tests demonstrated significance for bias in favour of smaller studies with positive effect sizes. While this may lend additional confidence to the overall non‐significant findings of this review given the possible over‐representation of positive studies, the significant presence of publication bias is still reason to view currently reported positive results with added scepticism, in particular the positive association demonstrated between FIS and self‐reported T2DM. Furthermore, it should be emphasized that the funnel‐plot‐asymmetry tests have low statistical power when analysing a small (<10) number of pooled studies.^90^ Therefore, although we aimed to increase the sensitivity by using both tests, it is possible that they may have simultaneously missed publication bias in some of our meta‐analyses.

## 5 CONCLUSIONS

This systematic review and meta‐analysis provides meaningful insights into the association between FIS and T2DM. The non‐significant associations between FIS and clinically determined T2DM and FBG or HbA1c found in this review suggest a need to revisit proposed relationships between FIS and mechanisms of diabetes risk. Additionally, our results indicate a need for additional works which investigate and report associations between FIS and T2DM in specific subpopulations which are currently lacking in the available literature. The finding of increased self‐reported T2DM among individuals with FIS underscores the unreliability of self‐reported health measures in lieu of direct, clinical measurements in the study of chronic disease in food insecure populations. Longitudinal studies on the chronic effects of FIS on risk of T2DM as well as additional paediatric studies on FIS and T2DM are warranted to better understand the possible effects of FIS on T2DM and glycaemic intolerance.

## CONFLICT OF INTERESTS

The authors have no conflicting or competing interests to declare.

## AUTHOR CONTRIBUTION

**Sourik Beltrán:** Conceptualization (equal); Data curation (equal); Formal analysis (equal); Project administration (supporting); Writing‐original draft (lead). **Daniel J. Arenas:** Conceptualization (equal); Data curation (equal); Formal analysis (equal); Methodology (lead); Writing‐review & editing (supporting). **Canada Montgomery:** Data curation (equal). **Marissa Pharel:** Data curation (supporting). **Itzel Lopez‐Hinojosa:** Data curation (supporting). **Horace M. DeLisser**: Project supervision (lead); Review of analyses (supporting); Editing the manuscript (lead).

## Supporting information

Supplementary Material

## Data Availability

There are no new unpublished data associated with this manuscript.
