# Supplementary material for: Food insecurity, type 2 diabetes, and hyperglycaemia: A systematic review and meta‐analysis
Source: Endocrinol Diabetes Metab. 2021 Nov 2;5(1):e00315. doi: 10.1002/edm2.315 (PMC8754242; doi:10.1002/edm2.315)
Supplement: Supplementary file 1 — Supplementary Material [file EDM2-5-e00315-s001.docx]

**Supporting Information**

**Food insecurity, type 2 diabetes, and hyperglycemia:**

**A systematic review and meta-analysis**

Sourik Beltrán, MD, MBE, Daniel J. Arenas, PhD, Marissa Pharel, BA

Canada Montgomery, BA, Itzel Lopez-Hinojosa, BA, Horace M. DeLisser, MD

Search Terms (pp. 2, 3)

Supplemental Tables, Tables S1-S11 (pp. 4-15)

Supplemental Figures, Figures S1-S14 (pp. 16-29)

Axis Tool Results and Discussion (p. 30)

References (pp. 31-36)

**Search Terms Utilized in the Systematic Search of the Literature**

| **Food Insecurity [12,726]** |  | **Cardiovascular Risk Factors** | | | |
| --- | --- | --- | --- | --- | --- |
|  |  |  |  |  |  |
| Food supply |  | **Diabetes mellitus Type 2 (205,860]** | **Metabolic syndrome (1,259]** | **Dyslipidemia (321,522)** | **Hypertension (443,154)** |
| Food supplies |  | Diabetes Mellitus, Noninsulin-dependent | Metabolic Syndromes | Dyslipidemias | Blood Pressure, High |
| Supplies, Food |  | Diabetes Mellitus, Ketosis-Resistant | Syndrome, Metabolic | Dyslipoproteinemias | Blood Pressures, High |
| Supply, Food |  | Diabetes Mellitus, Ketosis Resistant | Syndromes, Metabolic | Dyslipoproteinemia | High Blood Pressure |
| Food insecurities |  | Ketosis-Resistant Diabetes Mellitus | Metabolic Syndrome X | **Hyperlipidemia:** | High Blood Pressures |
| Insecurities, Food |  | Diabetes Mellitus, Non insulin Dependent | Insulin Resistance Syndrome X | Hyperlipemia | hypertension |
| Insecurity, Food |  | Diabetes Mellitus, Non-nsulin Dependent | Syndrome X, Metabolic | Hyperlipemias | Systolic pressure |
| Food Security |  | Diabetes Mellitus, Stable | Syndrome X, Insulin Resistance | Hyperlipidemia | Diastolic pressure |
| Security, Food |  | Stable Diabetes Mellitus | MEtabolic X Syndrome | Lipidemia | hypertensive |
| Food secure |  | Diabetes Mellitus, Type II | Syndrome, Metabolic X | Lipidemias |  |
| food insecure |  | NIDDM | X Syndrome, Metabolic | Lipemia |  |
|  |  | Diabetes Mellitus, Noninsulin Dependent | Dysmetabolic Syndrome X | Lipemias |  |
|  |  | Diabetes Mellitus, Maturity-Onset | Syndrome X, Dysmetabolic | lipids |  |
|  |  | Diabetes Mellitus, Maturity Onset | Reaven Syndrome X | triglycerides |  |
|  |  | Maturity-Onset Diabetes Mellitus | Syndrome X, Reaven | HDL |  |
|  |  | Maturity Onset Diabetes Mellitus | Metabolic Cardiovascular Syndrome | high-density lipoprotein |  |
|  |  | MODY | Cardiovascular Syndrome, Metabolic | high density lipoprotein |  |
|  |  | Diabetes Mellitus, Slow-Onset | Cardiovascular Syndromes, Metabolic | LDL |  |
|  |  | Diabetes Mellitus, Slow Onset | Syndrome, Metabolic Cardiovascular | low-density lipoprotein |  |
|  |  | Slow-Onset Diabetes Mellitus |  | low density lipoprotein |  |
|  |  | Type 2 Diabetes Mellitus |  | Hypertriglyceridemia |  |
|  |  | Noninsulin-Dependent Diabetes Mellitus |  |  |  |
|  |  | Noninsulin Dependent Diabetes Mellitus |  |  |  |
|  |  | Maturity-Onset Diabetes Mellitus |  |  |  |
|  |  | Diabetes, Maturity-Onset |  |  |  |
|  |  | Type 2 Diabetes Mellitus |  |  |  |
|  |  | Diabetes, Type 2 |  |  |  |
|  |  | Diabetes Mellitus, Adult-Onset |  |  |  |
|  |  | Adult-Onset Diabetes Mellitus |  |  |  |
|  |  | Diabetes Mellitus, Adult Onset |  |  |  |
|  |  | A1c |  |  |  |
|  |  | Hb A1c |  |  |  |
|  |  | HbA1c |  |  |  |
|  |  | Hemoglobin A1c |  |  |  |
|  |  | Hb1c |  |  |  |
|  |  | blood sugar |  |  |  |
|  |  | hyperglycemia |  |  |  |
|  |  | glucose tolerance test |  |  |  |
|  |  | glycemic index |  |  |  |
|  |  | insulin resistance |  |  |  |

**Supplemental Tables**

**Table S1.** Adult studies with sufficient primary data to retrieve or calculate the odds ratio for food insecurity with self-reported type 2 diabetes mellitus.

| **Study** | **Patients** | **Study Size** | **Diabetes Criteria** | **Results** | **Covariates** |
| --- | --- | --- | --- | --- | --- |
| Vozoris, 2003^1^ | Canada | 2,346 | Self-reported diabetes | AOR: 1.80 [1.2, 2.6]  Table 2 | age, education, and income |
| Stuff, 2006^2^ | US, Mississippi | 1,457 | Self-reported diabetes | OR: 1.72 [1.19, 2.48]*  Table 5 | n/a |
| Seligman, 2007^3^ | US NHANES 1999-2002 | 4,423 | Self-reported diabetes | AOR = 1.50 [0.77, 2.96] *,$$: FIS levels combined by RE model, Table 3 | age, gender, race/ethnicity, parity, income, family history, education, and BMI |
| Fitzgerald, 2011^4^ | US Latina women | 201 | Self-reported diabetes | OR: 2.18 [1.06, 4.49]*  Table 1 | n/a |
| Bomberg, 2018^5^ | Hunger in America | 49,751 | Self-reported diabetes | OR: 1.12 [1.06, 1.18]*  Table 1 | n/a |
| Perez-Escamilla, 2014^6^ | Mexico: Men | 13,411 | Self-reported diabetes | AOR = 1.22 [1.01, 1.46]  Table 3 | age, gender; education, area of residence, socioeconomic level, health care system affiliation (none, five public systems, private, ‘other’), and BMI |
|  | Mexico: Women | 18,909 |  | AOR = 1.46 [1.27, 1.69]  Table 3 |  |
|  | Combined | 32,320 |  | AOR = 1.35 [1.13, 1.61]  Table 3 |  |
| Bowen, 2015^7^ | USA (Chicago) | 153 | Self-reported diabetes | AOR: 2.56 [0.89, 7.39]  Table 3 | education, income, food stamps, housing subsidies, utilization of free meal sites, recent homeessness or incarceration. |
| Vaudin, 2015^8^ | USA, CC 2005–2006 | 566 | Self-reported diabetes | AOR: 1.87 [1.11, 3.14]  Table 2 | Age, gender, race, ethnicity, education, income, living arrangement, and region |
| Strings, 2016^9^ | US, California | 22,596 | Self-reported diabetes | OR: 1.22 [1.12, 1.34]*  Table 2 | n/a |
| BlueBird, 2017^10^ | US, Oklahoma | 513 | Self-reported diabetes | AOR: 1.40 [1.00, 1.99]  Table 2 | age, gender, study site, education, and income |
| Fernandes, 2018^11^ | Portugal | 1,626 | Self-reported diabetes | AOR: 1.76 [1.75, 1.78]  Table 6 | income, age group, gender, health region, and education |
| Ganhao, 2018^12^ | Lisbon 2015-2016 | 337 | Self-reported diabetes | AOR: 0.310 [0.130, 0.740]  Table 7 | gender, age, years of education, marital status, average household income |
| Garcia, 2018^13^ | US NHIS 2011-2014 | 2,150 | Self-reported diabetes | OR: 1.80 [1.62, 2.00]*  Table 1 | n/a |
| Helmick, 2018^14^ | US (N. Carolina & Virginia) | 930 | Self-reported diabetes | AOR: 1.51 [1.02, 2.23]  Table 3 | age, gender, race, education, income, employment, and marital status |
| Mendy, 2018^15^ | US, Mississippi | 5,870 | Self-reported diabetes | AOR: 1.30 [1.02, 1.65]  Table 3 | age, sex, race, education, health insurance, income |
| Venci, 2018^16^ | US NHS 2011 | 30,010 | Self-reported diabetes | AOR = 1.25 [1.10, 1.41]. *,$$: Table 4. | age, sex, race/ethnicity, marital status, education, income, weight, employment, children |
| Walker, 2018^17^ | US NHANES 2005-2014 | 27,218 | Self-reported diabetes | AOR = 1.66 [1.41, 1.95]  Table 1 | gender, age, race, education, marital status, income, survey year |
| Weigel,  2019^18^ | US Mexico  Border | 75 | Self-reported diabetes | AOR: 4.85 (1.59, 14.8)  Table 2 | age, sex, education, income |

*: Calculated by authors from primary data

$$: Data from different FIS levels was combined with RE model

**Table S2.** Adult studies with sufficient primary data to retrieve or calculate the odds ratio for food insecurity with type 2 diabetes mellitus as determined by fasting blood glucose measurements ≥126 mg/dL.

| **Study** | **Patients** | **Study Size** | **Diabetes Criteria** | **Results** | **Covariates** |
| --- | --- | --- | --- | --- | --- |
| Weigel, 2007^19^ | US Mexico border | 100 | Fasting blood glucose >126 mg/dl | AOR = 1.48 [0.56, 3.87]  Table 3 | age, sex, smoking and reproductive status |
| Yaemsiri, 2011^20^ | U.S. New York | 5,981 | Fasting blood glucose >126 mg/dl | AOR: 1.07 [0.83, 1.37] | age, sex, income, and BMI |
| Crews, 2014^21^ | HANDLS study | 1,239 | Fasting blood glucose >126 mg/dl | OR: 0.97 [0.72, 1.32]*  Table 3 | n/a |
| Shariff, 2014^22^ | Malaysia | 460 | Fasting blood glucose >126 mg/dl | AOR = 1.00 [0.69, 1.45]  Table 4 | age, ethnicity, urban/rural strata, education, employment and income per capita |
| Hasan- Ghomi, 2015^23^ | Iran | 400 | Fasting blood glucose >126 mg/dl | OR: 1.06 [0.72, 1.57]*  Table 2 | n/a |
| Najibi, 2019^24^ | Iran | 135 | Fasting blood glucose >126 mg/dl | AOR: 1.9 [1.13, 3.33].  Table 4 | age, marital status, employment, education, children, family size, income, menopause, and type of oil consumed |
| Weigel, 2019^18^ | US Mexico Border | 75 | Fasting blood glucose >126 mg/dl | AOR: 3.83 [1.60, 9.20]  Table 2 | age, sex, education, income, and BMI |

*: Calculated by authors from primary data

**Table S3.** Adult studies with sufficient primary data to retrieve or calculate the odds ratio for food insecurity with type 2 diabetes mellitus as determined by elevated hemoglobin A1c measurements.

| **Study** | **Patients** | **Study Size** | **Diabetes Criteria** | **Results** | **Covariates** |
| --- | --- | --- | --- | --- | --- |
| Gao, 2009^25^ | Puerto Ricans aged 45–75 y living in MA | 1,358 | HbA1c>7% | OR: 0.78 [0.55, 1.09]*  Table 1 | n/a |
| Terrell, 2009^26^ | US NHANES 1999-2004 | 15,199 | HbA1c>7% | AOR: 1.42 [1.04, 1.92]  Table 5 | age, sex, race/ethnicity, education, insurance status, marital status, incoem, BMI, smoking |
| Banerjee, 2017^27^ | US NHANES 1988-1994 | 2,320 | HbA1c>6.5% | OR: 1.03 [0.80, 1.31]*  Table 1 | n/a |
| Berkowitz, 2017^28^ | NHANES 2005 - 2012 | 3,540 | HbA1c>9% | OR: 1.93 [1.55, 2.40]*  Table 2 | n/a |
| Bergmans, 2019^29^ | US 2010–2014 Health and Retirement Study | 2,951 | HbA1c>7% | OR: 1.29 [1.05, 1.58]*  Table 1 | n/a |

*: Calculated by authors from primary data

**Table S4.** Adult studies with sufficient primary data to calculate standardized mean difference for food insecurity and fasting blood glucose.

| **Study** | **Patients** | **Study Size** | **Results** |
| --- | --- | --- | --- |
| Parker, 2010^30^ | NHANES 1999-2006 | 6,138 | g= 0.05 [-0.01, 0.12]. *,^: Table 1. ^: SD estimated from SE by assuming a normal distribution |
| Liu, 2015^31^ | US NHANES 2005-2010: Men | 2,742 | g = -0.05 [-0.13, 0.09]. *,^: Table 1. #: FIS levels combined by RE model |
|  | US NHANES 2005-2010: Women | 2,791 | g =0.01 [-0.08, 0.09]. *,^: Table 2.$$: FIS levels combined by RE model |
|  | US NHANES 2005-2010: Combined | 5,533 | g = -0.03 [-0.09, 0.03]. *,^: Calculated means and pooled standard deviations |
| Moghadam, 2016^32^ | Iran. 2015. | 243 | g = -0.05 [-0.32, 0.22]. *: Table 1 |
| Weigel, 2016^33^ | Ecuador | 269 | g = 0.02[-0.27, 0.31]. *,^: Table 4. |
| Bermudez-Millan, 2019^34^ | Latinos. CALMS-D study | 121 | g= 0.71 [0.32, 1.10]. *: Table 1. |
| Faramarzi, 2019^35^ | Iran | 151 | g = 0.09 [-0.52, 0.71]. *: Table 4 |

*: Calculated by authors from primary data

^: Combined different FIS levels by calculating mean and pooled standard deviation

**Table S5.** Adult studies with sufficient primary data to calculate standardized mean difference for food insecurity and hemoglobin A1c.

| **Study** | **Patients** | **Study Size** | **Results** |
| --- | --- | --- | --- |
| Holben, 2006^36^ | US Ohio | 738 | g = -0.18 [-0.35, -0.01] *,^: Combined different FIS levels by calculating mean and pooled standard deviation.  Table 5. |
| Ford, 2013^37^ | US NHANES 2003-2008 | 10,455 | g = 0.07 [0.03, 0.12]*  Table 1 |
| Moreno, 2015^38^ | Hispanic | 250 | g = 0.21 [-0.04, 0.45]*  Table 2 |
| Ippolito, 2016^39^ | Food Pantry participants across USA | 1,237 | g = 0.05 [-0.10, 0.20]*  Table 1 |
| Moghadam, 2016^32^ | Iranian adults | 243 | g = 0.08 [-0.19, 0.35]*  Table 1 |
| Shalowitz, 2017^40^ | USA adults | 336 | g = 0.23 [0.02, 0.45]*  Table 1 |
| Schroeder, 2018^41^ | USA Adults >65 | 2,968 | g = 0.25 [0.17, 0.34]*  Table 1 |
| Bermudez-Millan, 2019^34^ | Latinos. CALMS-D study | 121 | g = 0.13 [-0.25, 0.51]*  Table 1 |

*: Calculated by authors from primary data

^: Combined different FIS levels by calculating mean and pooled standard deviation

**Table S6.** Adult studies investigating beta coefficient of food insecurity with hemoglobin A1c.

| **Study** | **Patients** | **Study Size** | **Results** | **Covariates** | **BMI adjusted?** |
| --- | --- | --- | --- | --- | --- |
| Silverman, 2015^42^ | Peer-Aid 2011-2013. | 287 | Beta: 0.51  p<0.02  Table 3 | sex, age, race/ethnicity, language, education, marital status, BMI, insulin use, depression, diabetes distress and low medication adherence | yes |
| Smalls, 2015^43^ | US South East | 615 | Beta: 0.092, p>0.05  Table 4 | age, sex, education, income, marital status, and employment | no |
| Heerman, 2016^44^ | US Tennessee | 401 | Beta: 0.12 (0.01, 0.23) p<0.03  Table 3 | age, gender, race/ethnicity, education level, income, BMI and duration of diabetes. | yes |

**Table S7.** Adult OR studies which determined type 2 diabetes mellitus through means other than self-report, fasting blood glucose, or hemoglobin A1c.

| **Study** | **Patients** | **Study Size** | **Diabetes Criteria** | **Results** | **Covariates** |
| --- | --- | --- | --- | --- | --- |
| Wang, 2015^45^ | US Veterans. VACS 2002-2008 | 6,709 | EHR documentation of diabetes | OR 0.89 [0.78, 1.02]*  Table 1 | n/a |
| Tait, 2018^46^ | Canadian adults | 4,739 | EHR documentation of diabetes | AOR 2.04 [0.99, 4.26]  Table 2 | age, race, gender, income, BMI |
| Crews, 2014^21^ | NHANES 2003-2008 | 9,126 | Non-fasting blood glucose >200 mg/dl | OR: 1.07 [0.91, 1.25]*  Table 1 | n/a |

*: Calculated by authors from primary data

**Table S8.** Pediatric studies with sufficient primary data to calculate standardized mean difference for food insecurity and hemoglobin A1c.

| **Study** | **Patients** | **Study Size** | **Results** |
| --- | --- | --- | --- |
| Marjerrison, 2011^47^ | Australian children | 183 | g = 0.38 [0.03, 0.73]. *,^: Combined different FIS levels by calculating mean and pooled standard deviation .  Table 1 |
| Holben, 2015^48^ | US NHANES 1999-2006 | 7435 | g = 0.04 [-0.01, 0.09]. *,^  Table 2 |
| Lee, 2019^49^ | US NHANES 2003-2014 | 2662 | g = 0.10 [0.04, 0.17]. *,^  Table 2 |

*: Calculated by authors from primary data

^: Combined different FIS levels by calculating mean and pooled standard deviation

**Table S9.** Studies containing only adjusted odds ratios for food insecurity and T2DM determined by fasting blood glucose (FBG).

| **Study** | **Patients** | **Study Size** | **Diabetes Criteria** | **Results** | **Covariates** | **BMI adjusted?** |
| --- | --- | --- | --- | --- | --- | --- |
| Weigel, 2007^19^ | US Mexico border | 100 | FBG >126 mg/dl | AOR = 1.48 [0.56, 3.87]  Table 3 | age, sex, smoking and reproductive | no |
| Yaemsiri, 2011^20^ | U.S. New York | 5,981 | FBG >126 mg/dl | AOR: 1.07 [0.83, 1.37] | age, sex, income, and BMI | yes |
| Shariff, 2014^22^ | Malaysia | 460 | FBG >126 mg/dl | AOR = 1.00 [0.69, 1.45]  Table 4 | age, ethnicity, urban/rural strata, education, employment and income per capita | no |
| Najibi, 2019^24^ | Iran | 135 | FBG >126 mg/dl | AOR: 1.9 [1.13, 3.33].  Table 4 | age, marital status, employment status, education level, children, family size, children < 18-year-old, income, menopause, oil consumed | no |
| Weigel, 2019^18^ | US Mexico Border | 75 | FBG >126 mg/dl | AOR: 3.83 [1.60, 9.20]  Table 2 | age, sex, education, income, and BMI | yes |

**Table S10.** Studies containing only unadjusted odds ratios for food insecurity and HbA1c.

| **Study** | **Patients** | **Study Size** | **Diabetes Criteria** | **Results** |
| --- | --- | --- | --- | --- |
| Gao, 2009^25^ | Puerto Ricans aged 45–75 y living in MA | 1,358 | HbA1c>7% | OR: 0.78 [0.55, 1.09]*  Table 1 |
| Banerjee, 2017^27^ | US NHANES 1988-1994 | 2,320 | HbA1c>6.5% | OR: 1.03 [0.80, 1.31]*  Table 1 |
| Berkowitz, 2017^28^ | NHANES 2005 - 2012 | 3,540 | HbA1c>9% | OR: 1.93 [1.55, 2.40]*  Table 2 |
| Bergmans, 2019^29^ | U.S. 2010–2014 Health and Retirement Study | 2,951 | HbA1c>7% | OR: 1.29 [1.05, 1.58]*  Table 1 |

**Table S11.** Studies containing odds ratios for FIS and T2DM using HbA1c cut off at 7% only.

| **Study** | **Patients** | **Study Size** | **Diabetes Criteria** | **Results** | **Covariates** | **BMI adjusted?** |
| --- | --- | --- | --- | --- | --- | --- |
| Gao, 2009^25^ | Puerto Ricans aged 45–75 y living in MA | 1,358 | HbA1c>7% | OR: 0.78 [0.55, 1.09]*  Table 1 | n/a | n/a |
| Terrell, 2009^26^ | US NHANES 1999-2004 | 15,199 | HbA1c>7% | AOR: 1.42 [1.04, 1.92]  Table 5 | age, sex, race/ethnicity, education, insurance, marital status, poverty, BMI, smoking | yes |
| Bergmans, 2019^29^ | U.S. 2010–2014 Health & Retirement | 2,951 | HbA1c>7% | OR: 1.29 [1.05, 1.58]*  Table 1 | n/a | n/a |

**Supplemental Figures**

**Figure S1.** Flow-chart depicting results of the systematic literature search and study selection process.


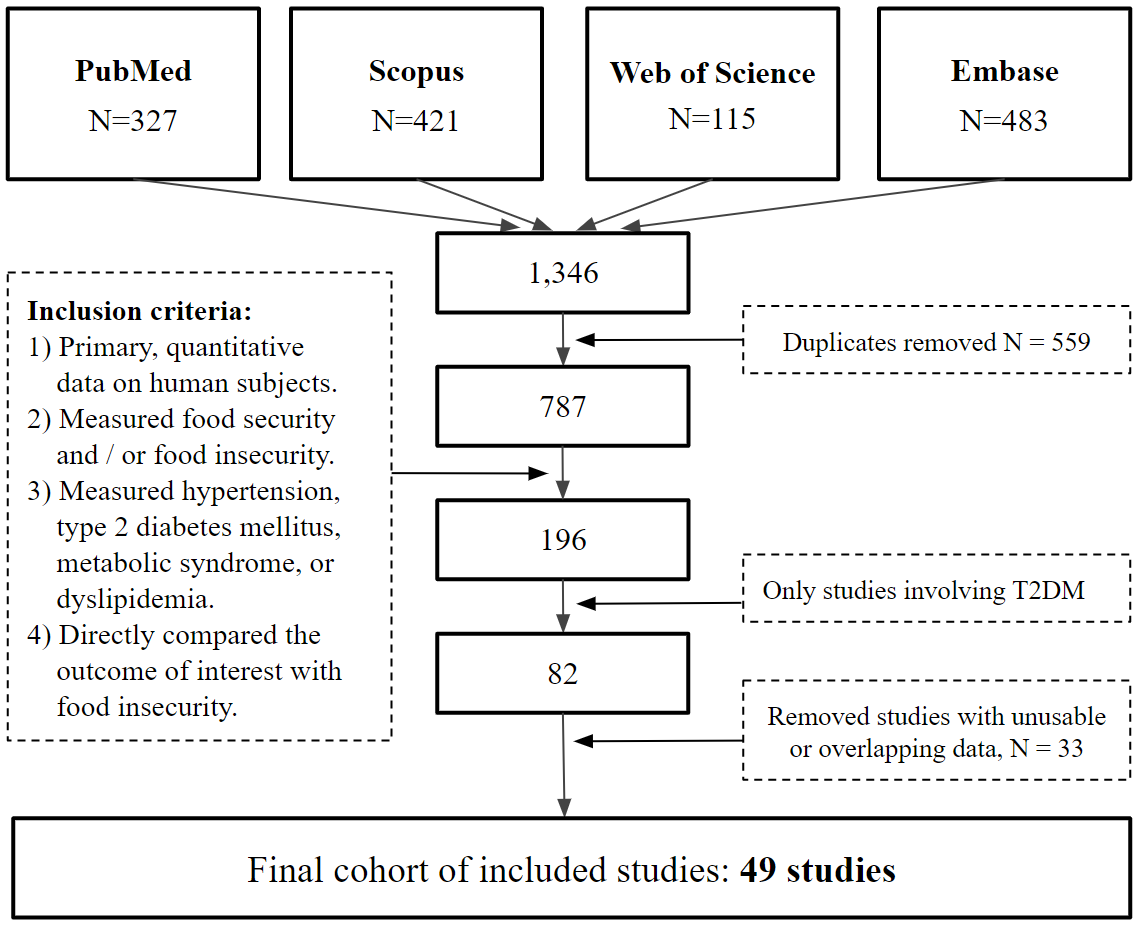


**Figure S2.** Funnel plot for meta-analysis of adult studies investigating the association between FIS and self-reported T2DM (See Figure 1 in main document). The Begg-Mazumdar rank correlation test indicated significant funnel-plot-asymmetry (p = 0.017); the Egger’s regression test was not significant (p = 0.497).


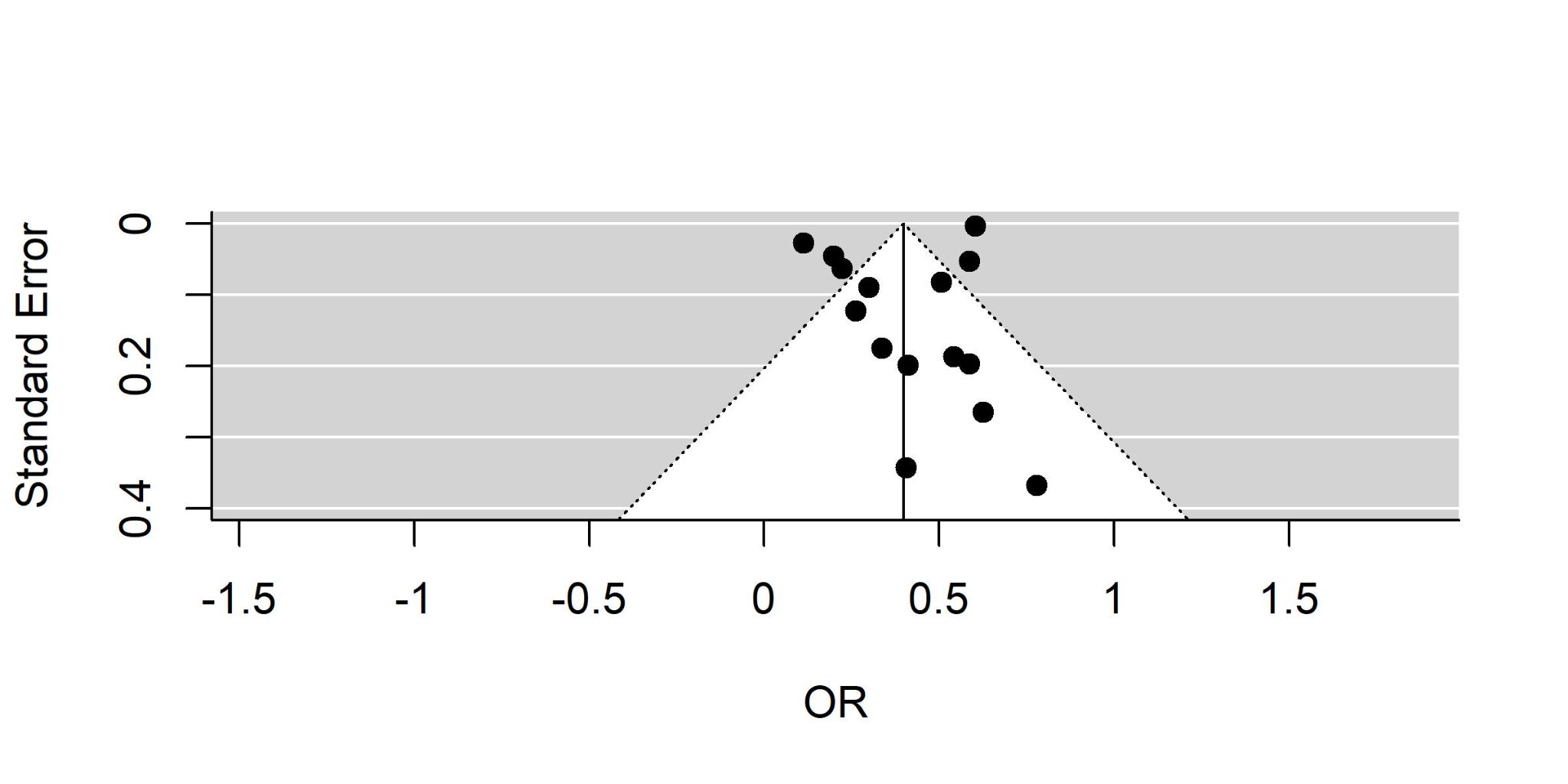


**Figure S3.** Funnel plot for meta-analysis of adult studies investigating FIS and T2DM as determined by FBG measurements (See Figure 2 in main manuscript). The Egger’s regression test indicated significant funnel-plot-asymmetry (p = 0.006); the Begg-Mazumdar rank correlation test was not significant (p = 0.136).


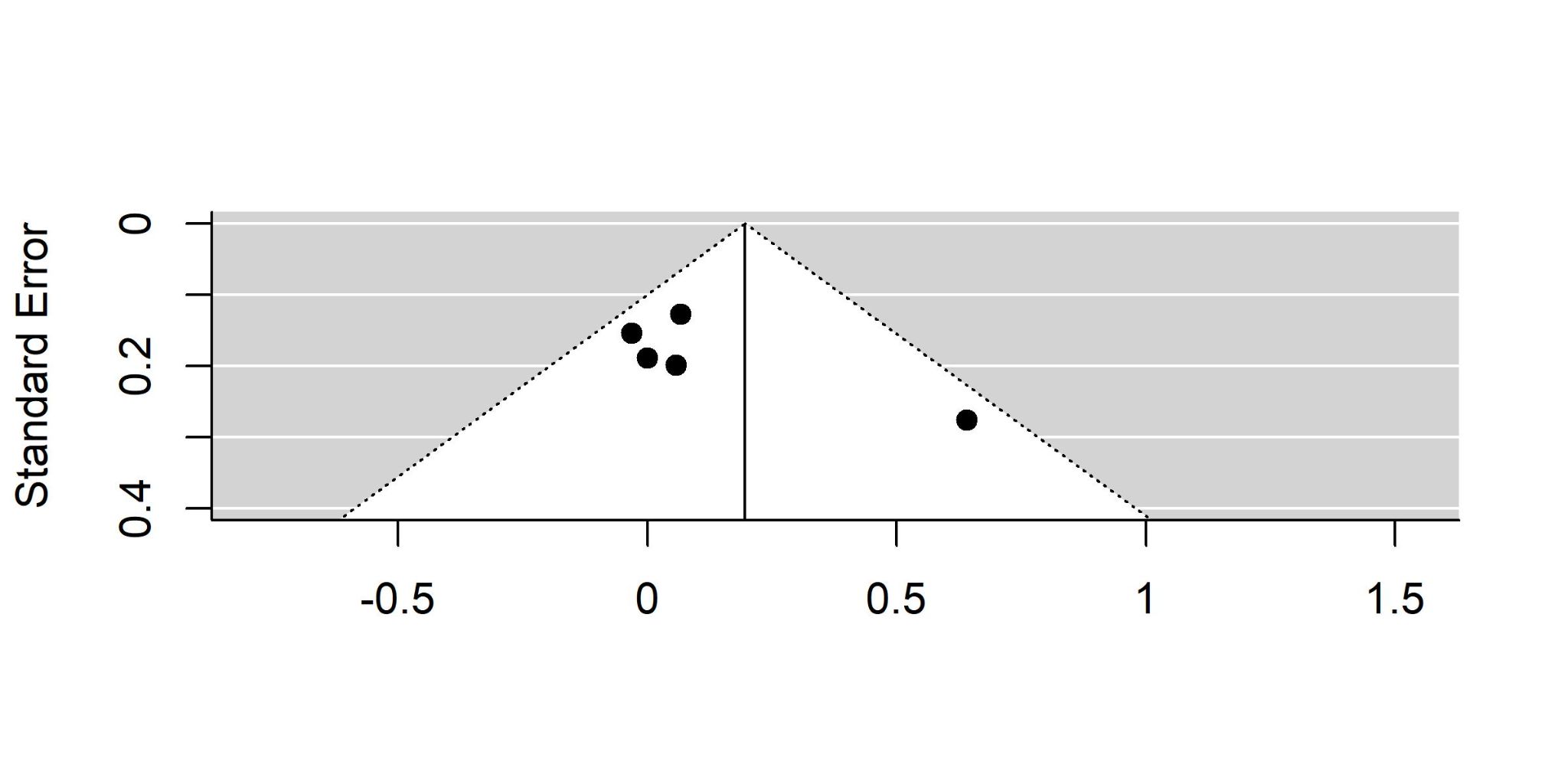


**Figure S4.** Funnel plot for meta-analysis of adult studies investigating FIS and T2DM as determined by HbA1c measurements (See Figure 3 in main manuscript). Both Egger’s regression test (p = 0.64) and the Begg-Mazumdar rank test (p = 0.82) were not significant for funnel-plot-asymmetry.


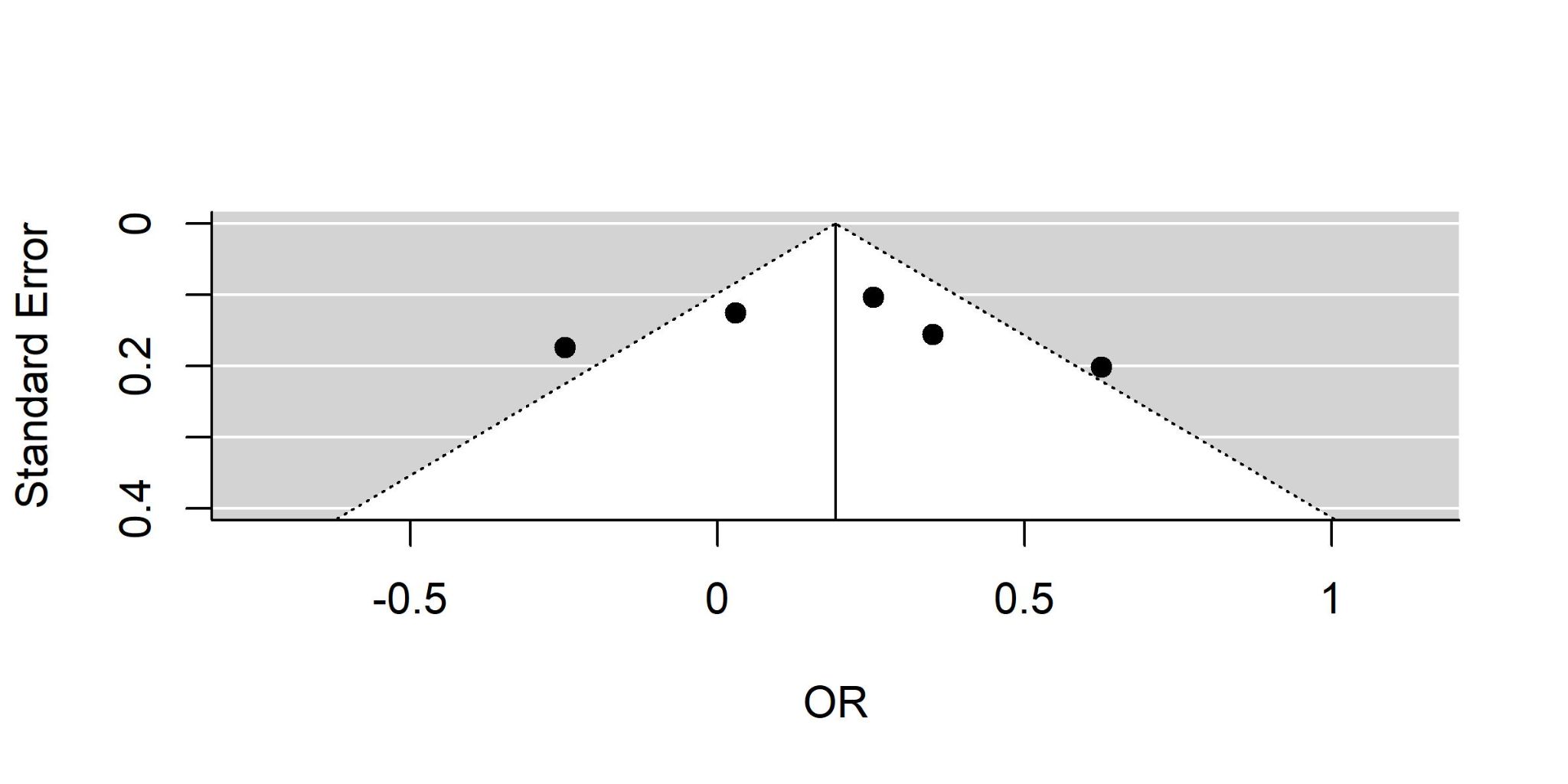


**Figure S5.** Funnel plot for meta-analysis of SMD for FIS and FBG among adult patients (See Figure 4 in main document). Both Egger’s regression test (p = 0.14) and the Begg-Mazumdar rank test (p = 0.27) were not significant for funnel-plot-asymmetry.


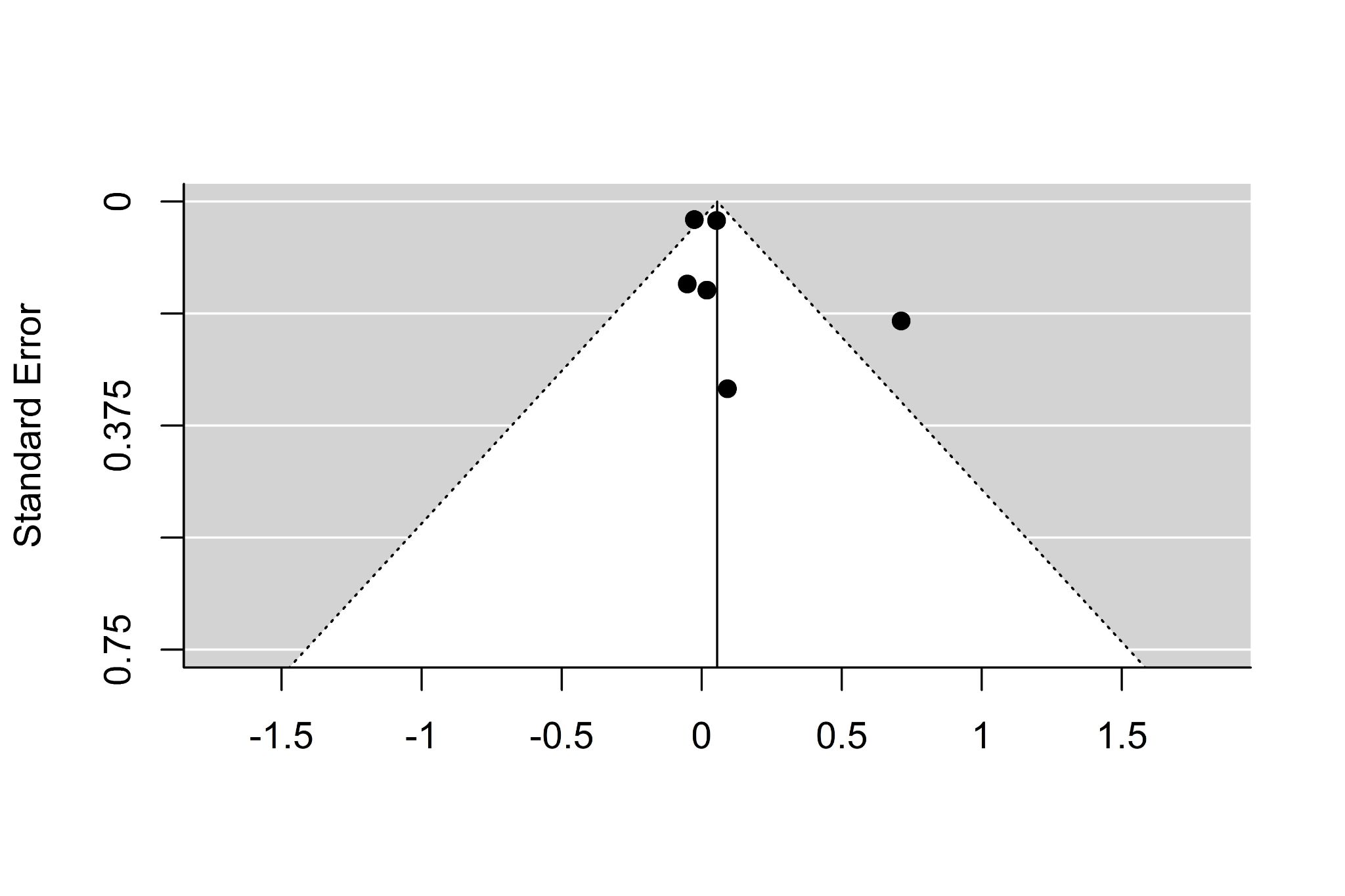


**Figure S6.** Funnel plot for meta-analysis of SMD for FIS and HbA1c among adult patients (See Figure 5 in main document). Both Egger’s regression test (p = 0.97) and the Begg-Mazumdar rank test (p = 1) were not significant for funnel-plot-asymmetry.
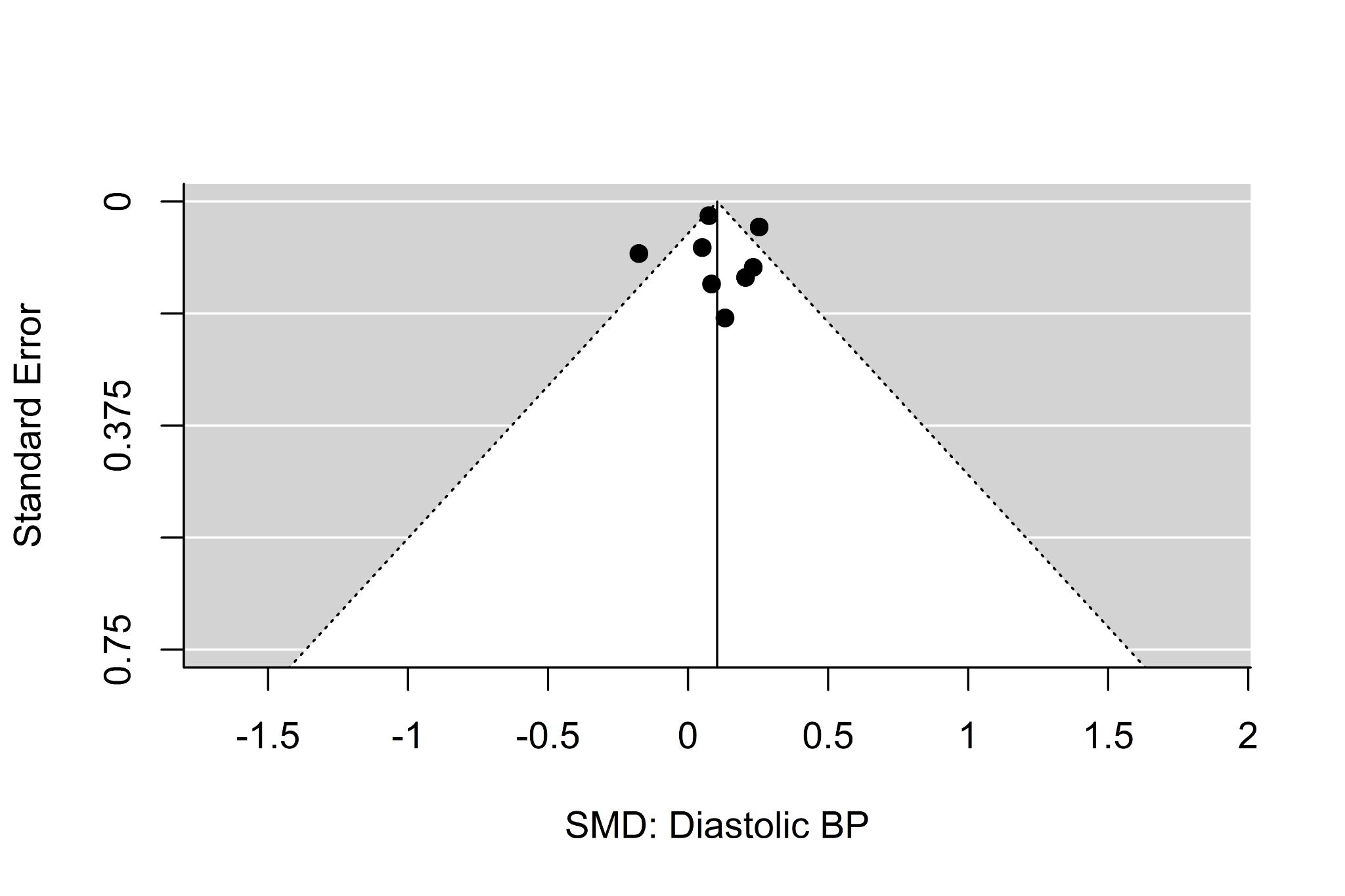


**Figure S7.** Results of meta-analysis for SMD of HbA1c among pediatric studies.


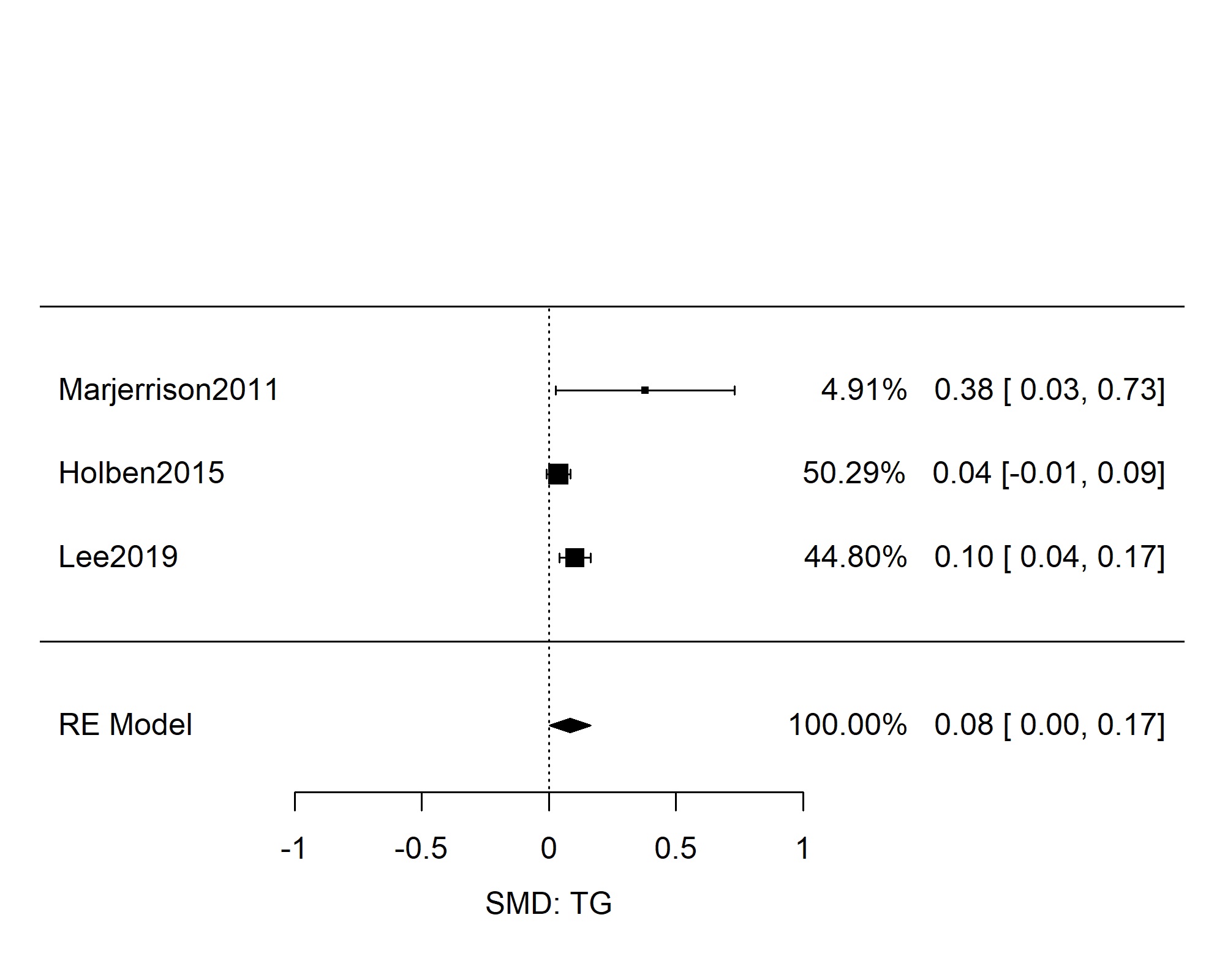


**Figure S8.** Funnel plot of meta-analysis for SMD of HbA1c among pediatric studies. Both Egger’s regression test (p = 0.053) and the Begg-Mazumdar rank test (p = 0.33) were not significant for funnel-plot-asymmetry.


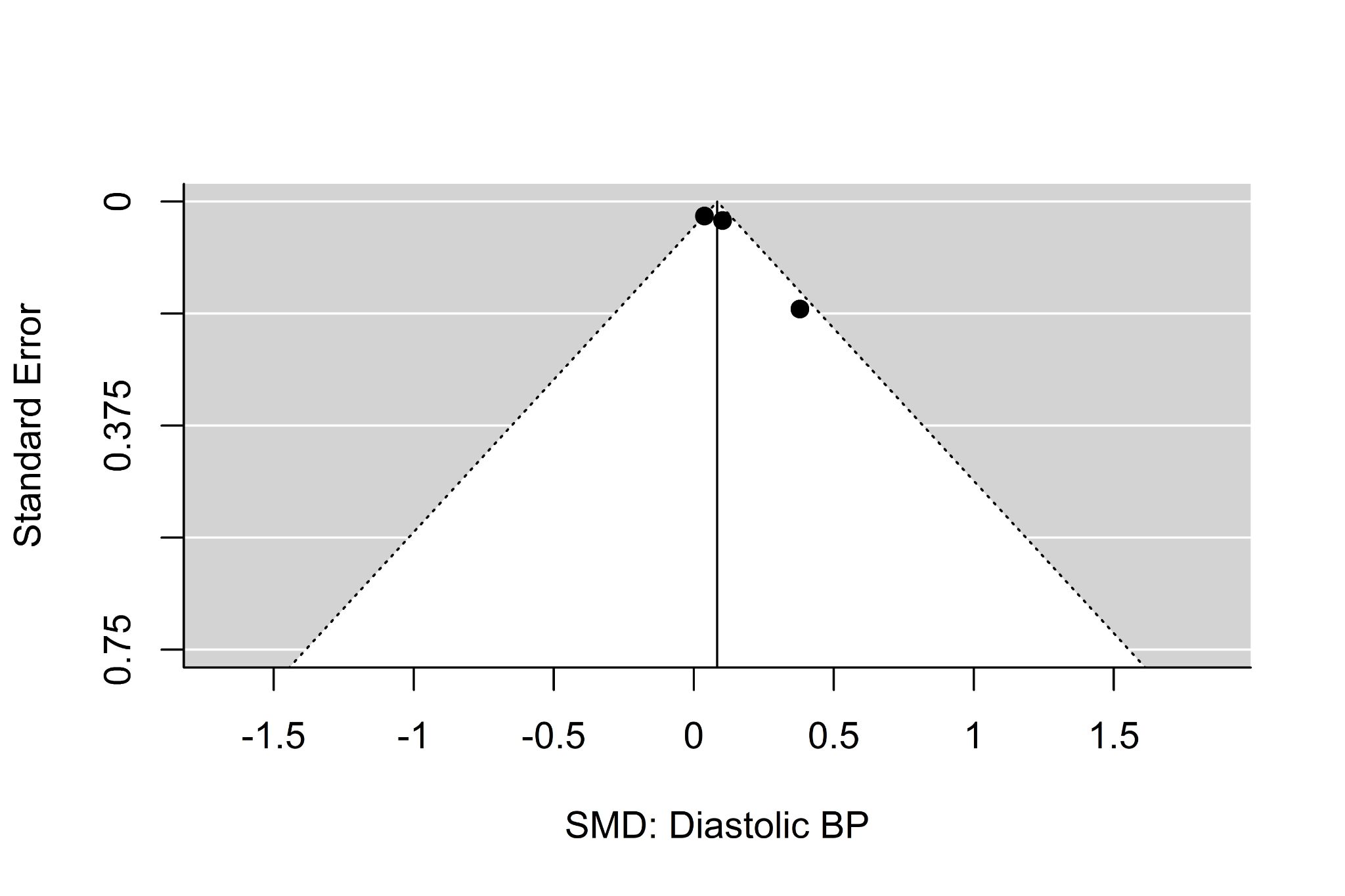


**Figure S9.** Results of subgroup meta-analysis of only adjusted odds ratios for FIS and T2DM determined by fasting blood glucose.
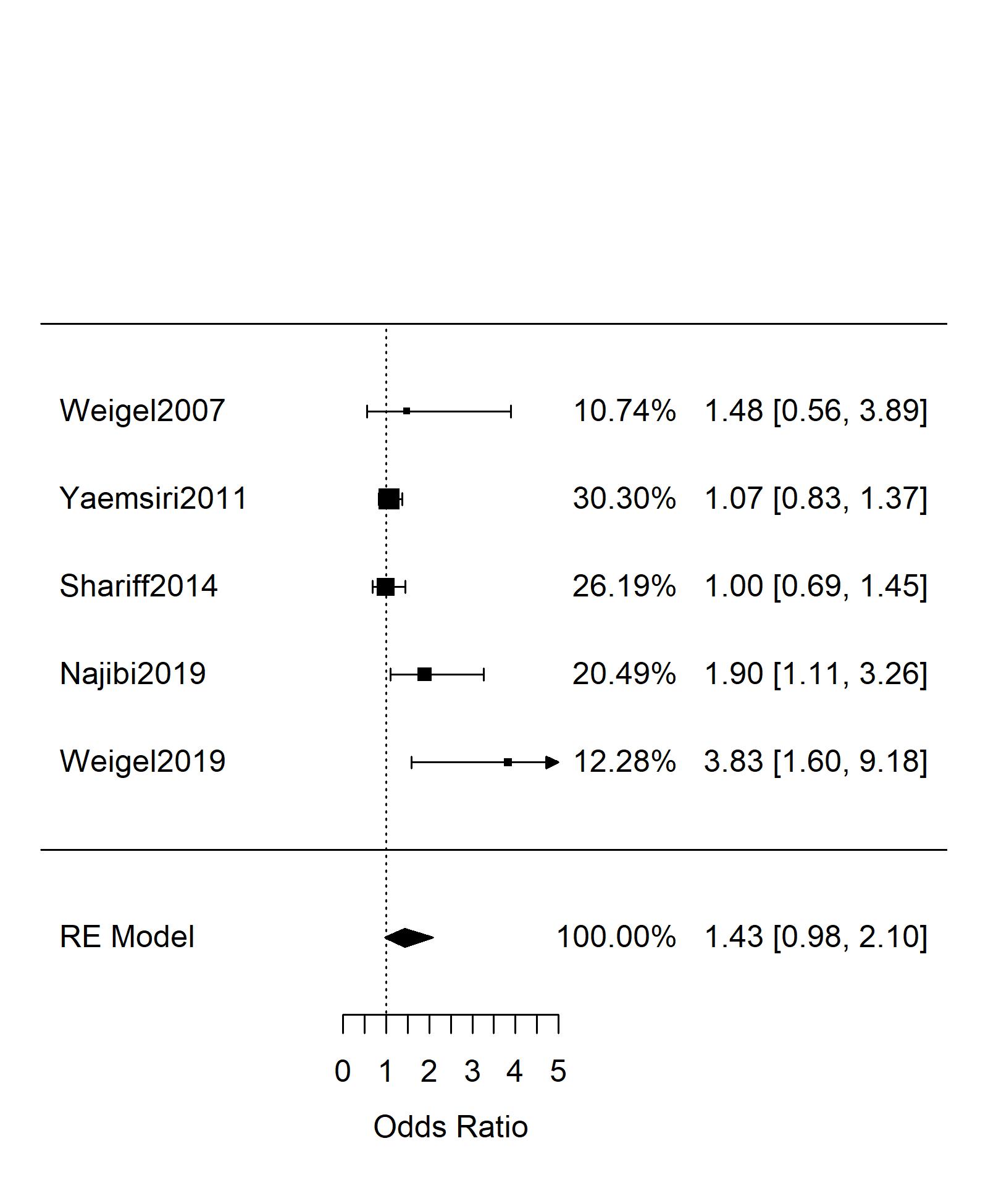


**Figure S10.** Funnel plot of subgroup meta-analysis of only adjusted odds ratios for FIS and T2DM determined by fasting blood glucose. The Egger’s regression test indicated significant funnel-plot-asymmetry (p = 0.02); the Begg-Mazumdar rank correlation test was not significant (p = 0.60).


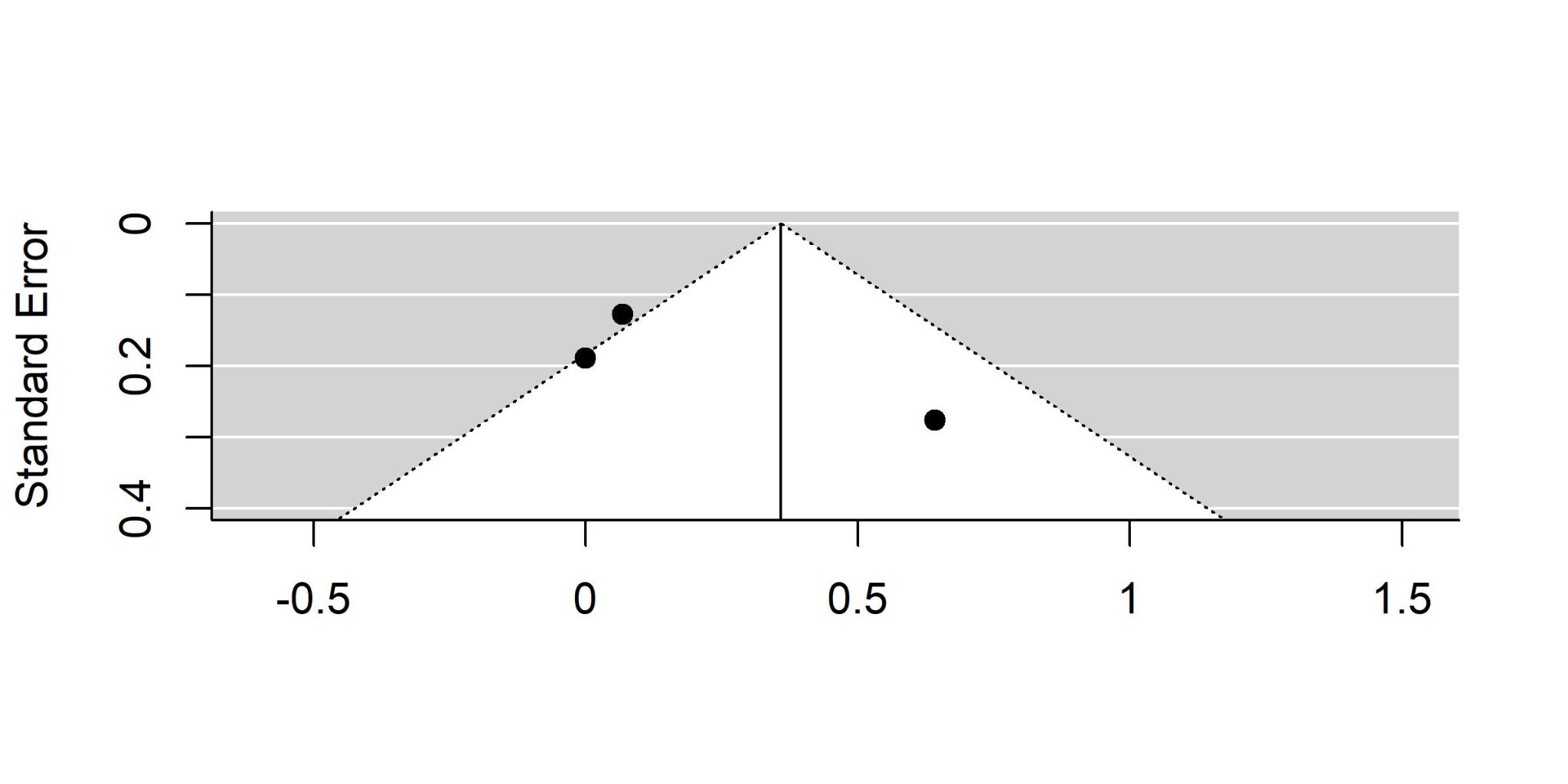


**Figure S11.** Results of subgroup meta-analysis of only unadjusted odds ratios for FIS and T2DM determined by HbA1c.


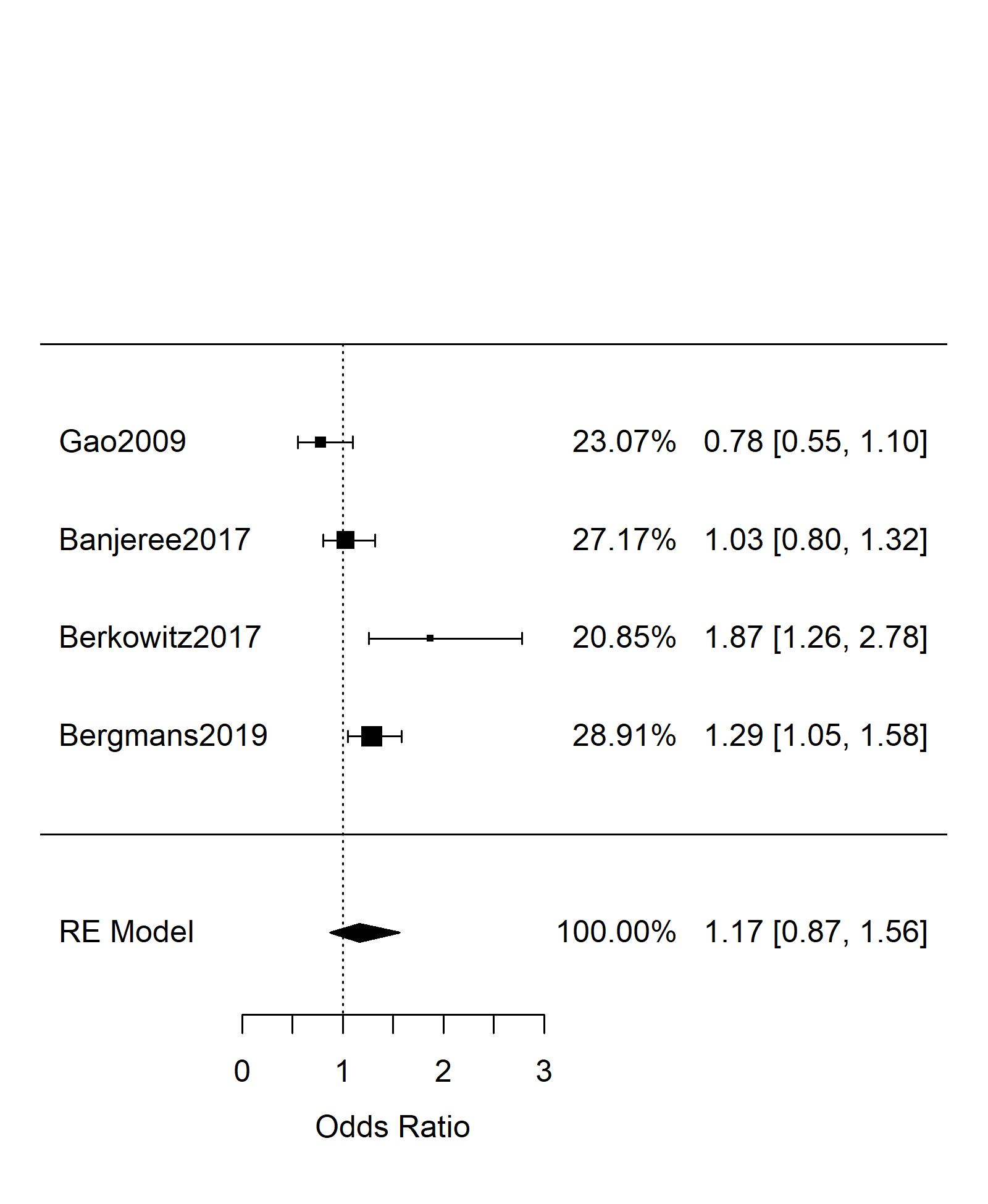


**Figure S12.** Funnel plot for subgroup meta-analysis of only unadjusted odds ratios for FIS and T2DM determined by HbA1c. Both Egger’s regression test (p = 0.710) and the Begg-Mazumdar rank test (p = 1) were not significant for funnel-plot-asymmetry.


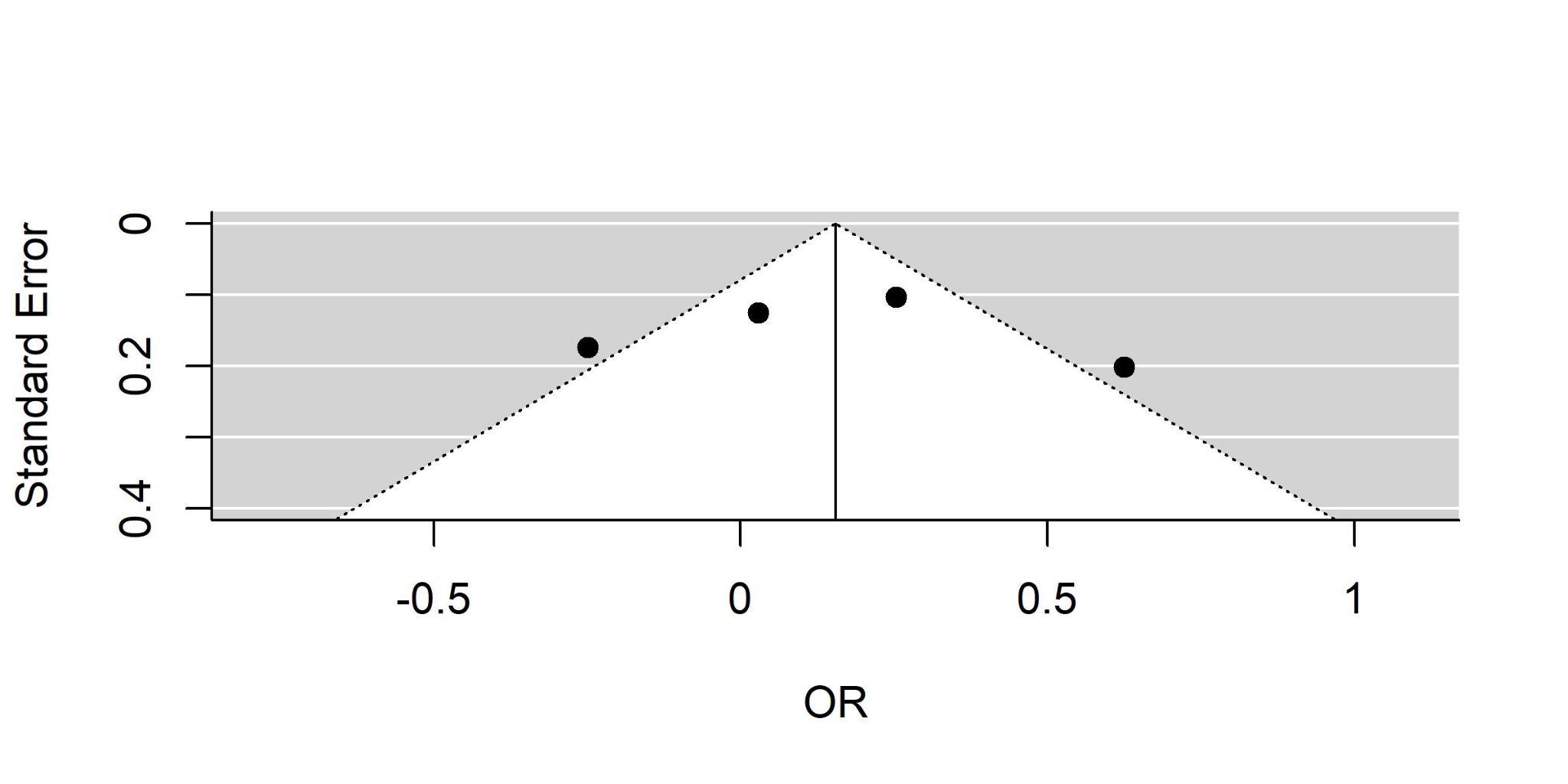


**Figure S13.** Results of subgroup meta-analysis of odds ratios for FIS and T2DM using HbA1c cut off at 7% only.


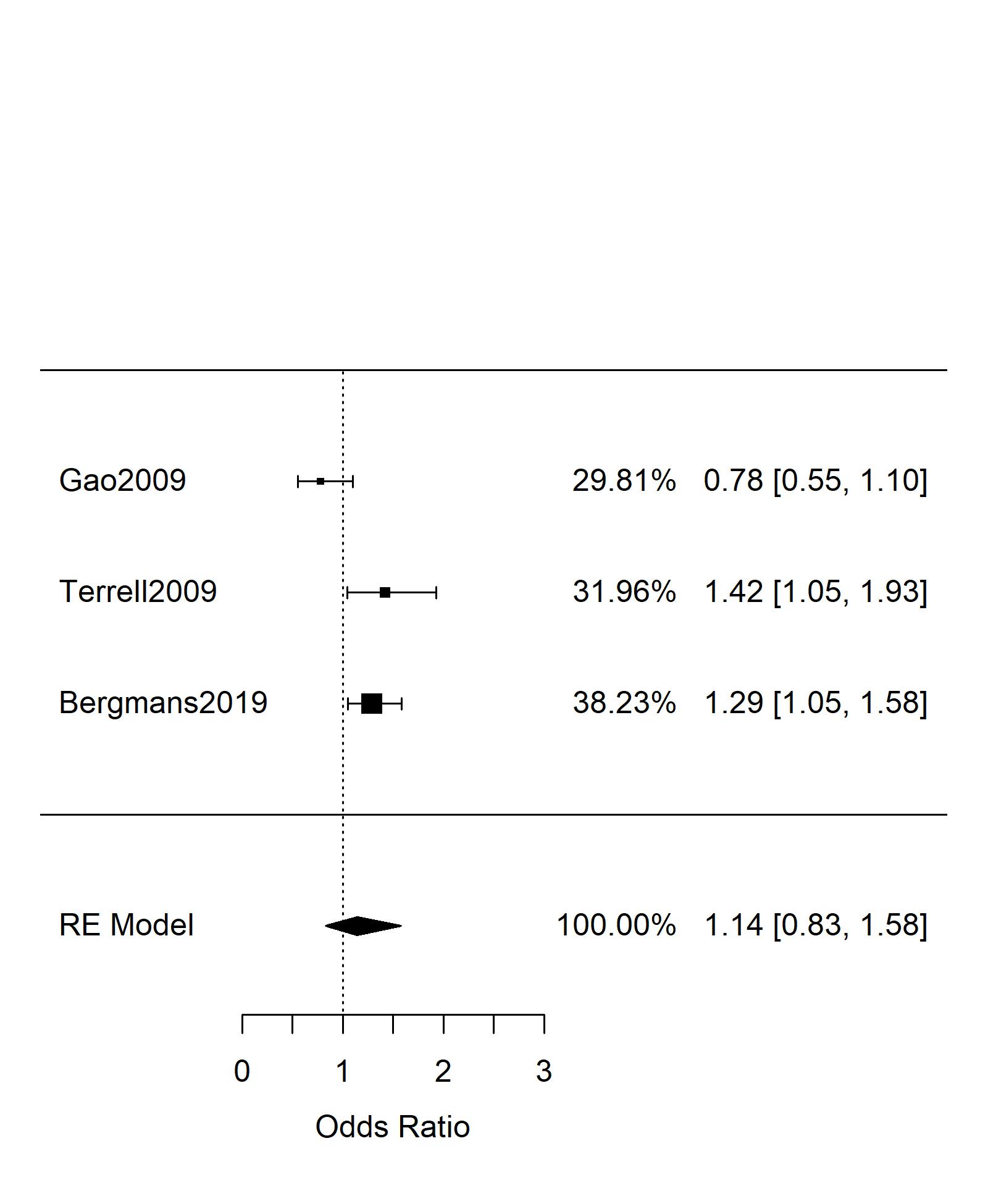


**Figure S14.** Funnel plot for subgroup meta-analysis of odds ratios for FIS and T2DM using HbA1c cut off at 7% only. Both Egger’s regression test (p = 0.50) and the Begg-Mazumdar rank test (p = 1) were not significant for funnel-plot-asymmetry.


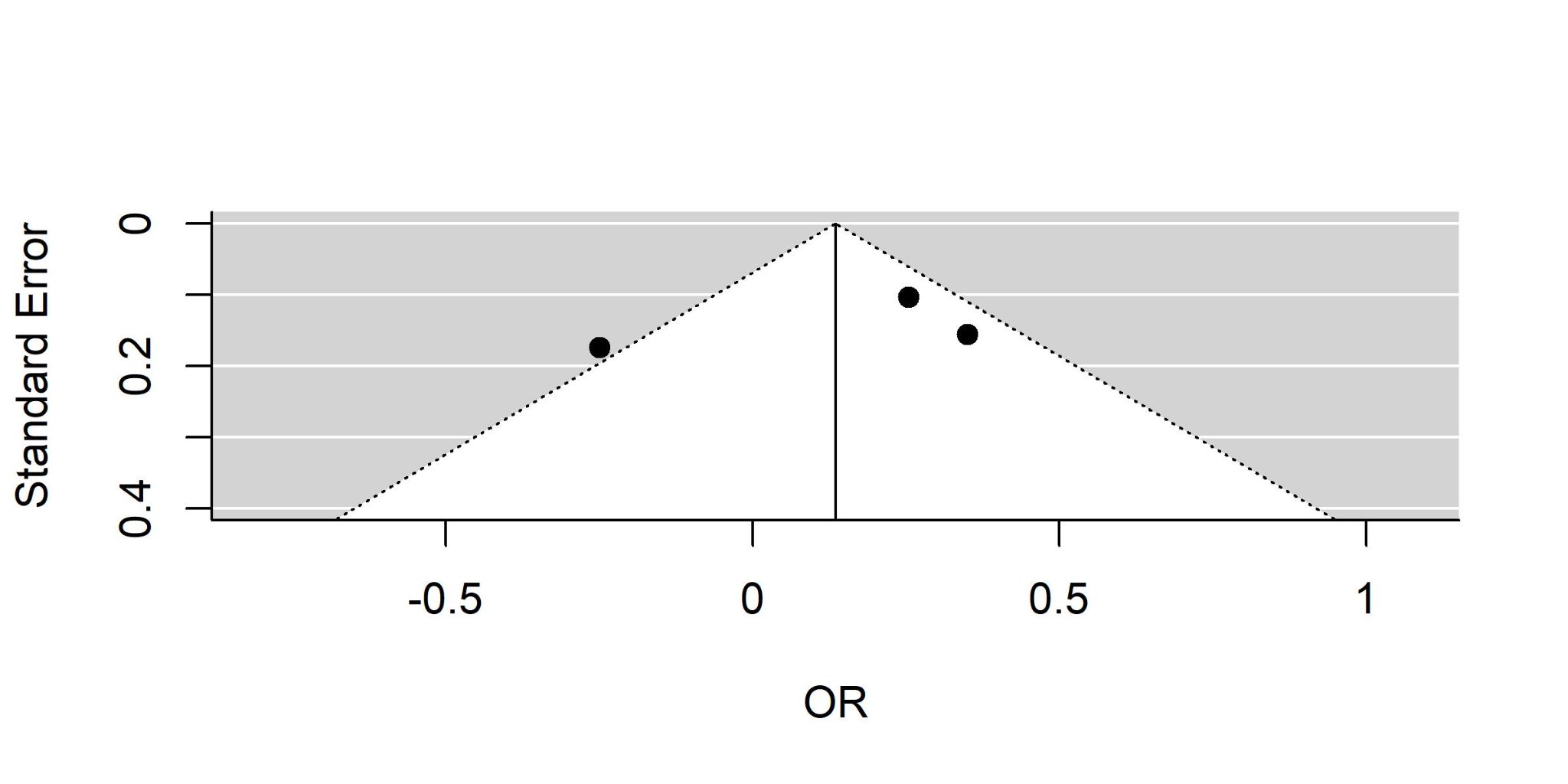


**AXIS Tool Results and Discussion**

Individual studies were evaluated by three authors by the AXIS appraisal tool for cross-sectional studies. From this analysis, it was found that only five of 49 studies reported explicit justifications for specific sample sizes used. However, this was seen as a reasonable limitation that would not warrant study exclusion as such information is not commonly reported in studies’ main text. Next, three studies (Walker, 2018; Terrell, 2009; Moghadam, 2016)^17,26,32^ were found to not explicitly report limitations. While this finding was concerning, we did not deem this sufficient to invalidate the data contributed by these three studies in meta-analysis. Finally, one study (Shalowitz, 2017) was found to not have explicitly stated that their study was approved by an appropriate ethics board.^40^ However, similar to the three studies which did not report limitations, we did not see this as sufficiently concerning to remove the study from our meta-analyses. No other concerning findings were raised in using the AXIS tool.

**References**

1. Vozoris NT, Tarasuk VS. Household food insufficiency is associated with poorer health. J Nutr. 2003;133: 120–126.
2. Stuff JE, McCabe-Sellers B, Casey PH, et al. Household Food Insecurity and Obesity, Chronic Disease, and Chronic Disease Risk Factors. Journal of Hunger and Environmental Nutrition. 2006;1(2); 43-62.
3. Seligman HK, Bindman AB, Vittinghoff E, et al. Food Insecurity is Associated with Diabetes Mellitus: Results from the National Health Examination and Nutrition Examination Survey (NHANES) 1999–2002. J Gen Internal Med. 2007;22:1018-1023.
4. Fitzgerald N, Hromi-Fiedler A, Segura-Perez S, Perez-Escamilla R. Food Insecurity is Related to Increased Risk of Type 2 Diabetes Among Latinas. Ethn Dis. 2011;21(3):328-334.
5. Bomberg EM, Neuhaus J, Hake MM, et al. Food Preferences and Coping Strategies among Diabetic and Nondiabetic Households Served by US Food Pantries. J of Hunger & Envir Nutr. 2018. 14:1-2, 4-17.
6. Pérez-Escamilla R, Villalpando S, Shamah-Levy T, Méndez-Gómez Humarán I. Household food insecurity, diabetes and hypertension among Mexican adults: Results from Ensanut 2012. Salud Pública México. 2014;56: s62–s70.
7. Bowen EA, Bowen SK, Barman-Adhikari A. Prevalence and covariates of food insecurity among residents of single-room occupancy housing in Chicago, IL, USA. Public Health Nutrition. 19(6):1122-1130.
8. Vaudin A, Sahyoun NR. Food Anxiety Is Associated with Poor Health Status Among Recently Hospital-Discharged Older Adults. J Nutr Gerontology and Geriatrics. 2015. 34(2):245-262.
9. Strings S, Ranchod YK, Laraia B, Nuru-Jeter A. Race and Sex Differences in the Association between Food Insecurity and Type 2 Diabetes. Ethnicity & Disease. 2016;26(2): 427-434.
10. Blue Bird Jernigan V, Wetherill MS, Hearod J, Jacob T, Salvatore AL, Cannady T, et al. Food insecurity and chronic diseases among American Indians in rural Oklahoma: The THRIVE study. Am J Public Health. 2017;107: 441–446.
11. Fernandes SG, Rodrigues AM, Nunes C, et al. Food Insecurity in Older Adults: Results From the Epidemiology of Chronic Disease Cohort Study 3. Front Med. 2018;5: 203.
12. Ganhao-Arranhado S, Paul C, Ramalho R, Pereira P. Food insecurity, weight and nutritional status among older adults attending senior centres in Lisbon. Arch of Gerontology and Geriatrics. 2018;78:81-88.
13. Garcia SP, Haddix A, Barnett K. Peer Reviewed: Incremental Health Care Costs Associated With Food Insecurity and Chronic Conditions Among Older Adults. Prev Chronic Dis. 2018;15.
14. Helmick M, Smith TM, Parks CA, Hill JL. Food insecurity increases odds of diabetes and hypertension, not obesity in medically underserved region. J Hunger Environ Nutr. 2020;15: 128–139.
15. Mendy VL, Vargas R, Cannon-Smith G, Payton M, Enkhmaa B, Zhang L. Food insecurity and cardiovascular disease risk factors among Mississippi adults. Int J Environ Res Public Health. 2018;15: 2016.
16. Venci BJ, Lee S-Y. Functional limitation and chronic diseases are associated with food insecurity among US adults. Ann Epidemiol. 2018;28: 182–188.
17. Walker RJ, Grusnick J, Garacci E, et al. Trends in Food Insecurity in the USA for Individuals with Prediabetes, Undiagnosed Diabetes, and Diagnosed Diabetes. J Gen Intern Med. 2018. 34(1);33-35.
18. Weigel MM, Armijos RX. Food insecurity, Cardiometabolic health, and health care in US-Mexico border immigrant adults: An exploratory study. J Immigr Minor Health. 2019;21: 1085–1094.
19. Weigel MM, Armijos RX, Hall YP, Ramirez Y, Orozco R. The household food insecurity and health outcomes of US–Mexico border migrant and seasonal farmworkers. J Immigr Minor Health. 2007;9: 157–169.
20. Yaemsiri S, Olson CE, He K, Kerker BD. Food concern and its associations with obesity and diabetes among lower-income New Yorkers. Public Health Nutrition. 2011. 15(1):39-47.
21. Crews DC, Kuczmarski MF, Grubbs V, et al. Effect of Food Insecurity on Chronic Kidney Disease in Lower-Income Americans. Am J Nephrol. 2014;39:27-35.
22. Shariff ZM, Sulaiman N, Jalil RA, Yen WC, Yaw YH, Mohd Taib MN, et al. Food insecurity and the metabolic syndrome among women from low income communities in Malaysia. Asia Pac J Clin Nutr. 2014;23: 138.
23. Hasan-Ghomi M, Ejtahed HS, Mirmiran P, et al. Relationship of Food Security with Type 2 Diabetes and Its Risk Factors in Tehranian Adults. Int J Prev Med. 2015;6:98.
24. Najibi N, Firoozi R, Shahrezaee S. Food insecurity is an important risk factor for type 2 diabetes: a case-control study of new referrals to the University clinics, Shiraz, Southern Iran. BMC Public Health. 2019;19:885.
25. Gao X, Scott T, Falcon LM, et al. Food insecurity and cognitive function in Puerto Rican adults. Am J Clin Nutr. 2009;89:1197-1203.
26. Terrell A. Is food insecurity associated with chronic disease and chronic disease control? Ethn Dis. 2009;19.
27. Banerjee T, Crews DC, Wesson DE, Dharmarajan S, Saran R, Burrows NR, et al. Food insecurity, CKD, and subsequent ESRD in US adults. Am J Kidney Dis. 2017;70: 38–47.
28. Berkowitz SA, Berkowitz TS, Meigs JB, Wexler DJ. Trends in food insecurity for adults with cardiometabolic disease in the United States: 2005–2012. PloS One. 2017;12.
29. Bergmans RS, Zivin K, Mezuk B. Depression, food insecurity and diabetic morbidity: Evidence from the Health and Retirement Study. J Psychosomatic Research. 2019;117: 22-29.
30. Parker ED, Widome R, Nettleton JA, Pereira MA. Food security and metabolic syndrome in US adults and adolescents: findings from the National Health and Nutrition Examination Survey, 1999–2006. Ann Epidemiol. 2010;20: 364–370.
31. Liu J, Mark YM, Berkowitz SA, et al. Gender differences in the association between food insecurity and insulin resistance among U.S. adults: National Health and Nutrition Examination Survey, 2005-2010. Annals of Epidemiology. 2015;25: 643-648.
32. Moghadam S, Javadi M, Mohammadpooral A. Relationship between Food Security with Sugar Level and Blood Pressure in Diabetes Type 2 in Tehran. Electron Physician. 2016;8: 3398.
33. Weigel MM, Armijos RX, Racines M, Cevallos W, Castro NP. Association of household food insecurity with the mental and physical health of low-income urban Ecuadorian women with children. J Environ Public Health. 2016;2016.
34. Bermudez-Millan A, Perez-Escamilla R, Segura-Perez S, et al. Psychological Distress Mediates the Association between Food Insecurity and Suboptimal Sleep Quality in Latinos with Type 2 Diabetes Mellitus. J Nutr. 2016;146(10):2051-2057.
35. Faramarzi E, Somi M, Ostadrahimi A, Dastgiri S, Nahand MG, Jafarabadi MA, et al. Association between food insecurity and metabolic syndrome in North West of Iran: Azar Cohort study. J Cardiovasc Thorac Res. 2019;11: 196.
36. Holben DH, Pheley AM. Peer reviewed: Diabetes risk and obesity in food-insecure households in rural Appalachian Ohio. Prev Chronic Dis. 2006;3.
37. Ford ES. Food security and cardiovascular disease risk among adults in the United States: findings from the National Health and Nutrition Examination Survey, 2003–2008. Prev Chronic Dis. 2013;10: E202–E202.
38. Moreno G, Morales LS, Isiordia M, de Jaimes FN, Tseng C-H, Noguera C, et al. Latinos with diabetes and food insecurity in an agricultural community. Med Care. 2015;53: 423.
39. Ippolito MM, Lyles CR, Prendergast K, et al. Food insecurity and diabetes self-management among food pantry clients. Public Health Nutrition. 2016;20(1): 183-189.
40. Shalowitz MU, Eng JS, McKinney CO, Krohn J, Lapin B, Wang CH, et al. Food security is related to adult type 2 diabetes control over time in a United States safety net primary care clinic population. Nutr Diabetes. 2017;7: e277–e277.
41. Schroeder EB, Zeng C, Sterrett AT, Kimpo TK, Paolino AR, Steiner JF. The longitudinal relationship between food insecurity in older adults with diabetes and emergency department visits, hospitalizations, hemoglobin A1c, and medication adherence. J Diabetes Complications. 2019;33: 289–295.
42. Silverman J, Krieger J, Kiefer M, et al. The Relationship Between Food Insecurity and Depression, Diabetes Distress and Medication Adherence Among Low-Income Patients with Poorly-Controlled Diabetes. J Gen Intern Med. 2015;30(10):1476-1480.
43. Smalls BL, Gregory CM, Zoller JS, Egede LE. Assessing the relationship between neighborhood factors and diabetes related health outcomes and self-care behaviors. BMC Health Services Research. 2015;15: 445.
44. Heerman W, Wallston KA, OSborn CY, et al. Research: Educational and psychological aspects Food insecurity is associated with diabetes self-care behaviours and glycaemic control. Diabet Med. 2016;33(6): 844-850.
45. Wang EA, McGinnis KA, Goulet J, Bryant K, Gibert C, Leaf DA, et al. Food insecurity and health: data from the Veterans Aging Cohort Study. Public Health Rep. 2015;130: 261–268.
46. Tait CA, L'abbe MR, Smith PM, Rosella LC. The association between food insecurity and incident type 2 diabetes in Canada: A population-based cohort study. 2018;13(5): e0195962.
47. Marjerrison S, Cummings EA, Glanville T, et al. Prevalance and Associations of Food Insecurity in Children with Diabetes Mellitus. J of Pediatrics. 2011;158(4): 607-611.
48. Holben DH, Taylor CA. Food Insecurity and Its Association With Central Obesity and Other Markers of Metabolic Syndrome Among Persons Aged 12 to 18 Years in the United States. J Am Osteopath Assoc. 2015;115(9): 536-543.
49. Lee AM, Scharf RJ, Filipp SL, Gurka MJ, DeBoer MD. Food Insecurity Is Associated with Prediabetes Risk Among US Adolescents, NHANES 2003–2014. Metab Syndr Relat Disord. 2019;17: 347–354.
